# A subpopulation of CD146^+^ macrophages enhances antitumor immunity by activating the NLRP3 inflammasome

**DOI:** 10.1038/s41423-023-01047-4

**Published:** 2023-06-12

**Authors:** Lin Jing, Yunhe An, Tanxi Cai, Jianquan Xiang, Baoming Li, Jiang Guo, Xinran Ma, Ling Wei, Yanjie Tian, Xiaoyan Cheng, Xuehui Chen, Zheng Liu, Jing Feng, Fuquan Yang, Xiyun Yan, Hongxia Duan

**Affiliations:** 1 Key Laboratory of Protein and Peptide Pharmaceutical, Institute of Biophysics, Chinese Academy of Sciences, Beijing, 100101 China; 2 Institute of Analysis and Testing, Beijing Academy of Science and Technology (Beijing Center for Physical and Chemical Analysis), No. 7 Fengxian Middle Street, Haidian District, Beijing, 100094 China; 3 Sino-Danish College, University of Chinese Academy of Sciences, Beijing, 100049 China; 4 Laboratory of Proteomics, Institute of Biophysics, Chinese Academy of Sciences, Beijing, China; 5 College of Life Sciences, University of Chinese Academy of Sciences, Beijing, 100049 China; 6 Department of Interventional Oncology, Beijing Ditan Hospital, Capital Medical University, No. 8 Jingshun East Street, Chaoyang District, Beijing, 100015 China; 7 Joint Laboratory of Nanozymes in Zhengzhou University, School of Basic Medical Sciences, Zhengzhou University, Zhengzhou, 450001 China

**Keywords:** Tumor-associated macrophages, CD146, Inflammasome, TMEM176B, Tumor immunotherapy, Tumour immunology, Immunotherapy

## Abstract

As one of the main tumor-infiltrating immune cell types, tumor-associated macrophages (TAMs) determine the efficacy of immunotherapy. However, limited knowledge about their phenotypically and functionally heterogeneous nature restricts their application in tumor immunotherapy. In this study, we identified a subpopulation of CD146^+^ TAMs that exerted antitumor activity in both human samples and animal models. CD146 expression in TAMs was negatively controlled by STAT3 signaling. Reducing this population of TAMs promoted tumor development by facilitating myeloid-derived suppressor cell recruitment via activation of JNK signaling. Interestingly, CD146 was involved in the NLRP3 inflammasome-mediated activation of macrophages in the tumor microenvironment, partially by inhibiting transmembrane protein 176B (TMEM176B), an immunoregulatory cation channel. Treatment with a TMEM176B inhibitor enhanced the antitumor activity of CD146^+^ TAMs. These data reveal a crucial antitumor role of CD146^+^ TAMs and highlight the promising immunotherapeutic approach of inhibiting CD146 and TMEM176B.

## Introduction

Immunotherapy has achieved astounding success in the clinical treatment of cancer patients [1]. However, the immunotherapy response rate is still low and many patients do not respond to this treatment [2].

The composition of immune cells in the tumor microenvironment (TME) often determines the efficacy of a specific therapy, especially tumor immunotherapy [2]. Tumor-associated macrophages (TAMs), representing up to 50% of immune cells in the TME [3, 4], play opposing roles in tumorigenesis. In the early stage, TAMs exhibit an antitumor (or M1-like) phenotype, which allows them to clear tumor cells. As tumorigenesis progresses, however, TAMs switch to a pro-tumor (or M2-like) phenotype and help shape a suitable microenvironment for tumor growth, survival, metastasis and angiogenesis [5]. TAMs can also reduce the effectiveness of immunotherapy via their immunosuppressive function [6]. Mounting evidence shows that targeting TAMs in personalized medicine can enhance the antitumor effects of chemotherapy and immunotherapy [3, 7, 8]. However, knowledge about the phenotypically and functionally heterogeneous nature of TAMs is limited, and further studies are needed to elucidate their phenotypes and functions to develop new strategies for cancer treatment.

Cluster of differentiation 146 (CD146), also known as melanoma cell adhesion molecule (MCAM) or mucin 18 (MUC18), is a member of the immunoglobulin (Ig) superfamily and is reported to be involved in various pathological conditions, including cancer and chronic inflammatory diseases [9–13]. Recent studies have shown that CD146 also regulates the functions of immune cells such as T cells and macrophages [11, 14, 15]. Under normal physiological conditions, the expression of CD146 in macrophages is low; under inflammatory conditions, CD146 is significantly upregulated. Consistently, CD146 has been positively correlated with pulmonary inflammation [16]. Our previous study also found that CD146 is highly expressed in macrophages in atherosclerotic plaques, promoting low-density lipoprotein internalization and macrophage foam cell formation, thereby accelerating plaque necrosis and disease progression [14]. In our recent study, we showed that CD146 regulates proinflammatory processes in high-fat diet-induced obese mice [17]. Therefore, CD146 might control macrophage proinflammatory processes in a variety of inflammatory conditions. Considering the similarity of chronic inflammation in cancer, we hypothesized that CD146 was associated with the proinflammatory polarization of TAMs.

Therefore, we investigated CD146 expression in TAMs derived from human tumor samples and cancer model animals and elucidated the function of CD146^+^ TAMs in tumor development. Our findings suggested that CD146^+^ TAMs are located at the margins of tumors and play an antitumor role. Deleting this subtype accelerated tumor growth by reducing T-cell infiltration and increasing myeloid-derived suppressor cell (MDSC) count. In particular, CD146 deletion promoted the c-*Jun* kinase (*JNK*) signaling pathway, resulting in higher expression levels of transmembrane protein 176B (TMEM176B), an immunoregulatory cation channel [18], and less activation of interleukin-1β (IL-1β), suggesting inhibition of NLRP3 inflammasome activation. Our data also showed that the combination of anti-CD146 + TMEM176B inhibitor therapy enhanced antitumor efficiency. The findings of this study provide new insights into the phenotypically and functionally heterogeneous nature of TAMs and assist in the development of new strategies for cancer immunotherapy.

## Results

### CD146 was expressed on a subset of macrophages in the tumor microenvironment

**Fig. 1:**
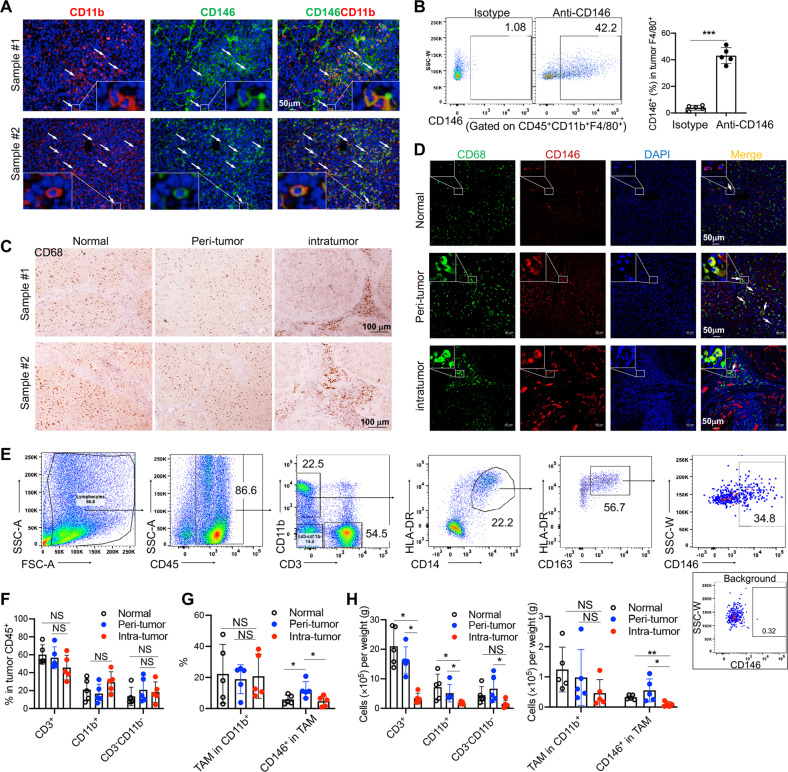
CD146 was expressed on a subset of macrophages in the TME. **A** Immunostaining (DAPI, nuclear staining) of CD11b (red) and CD146 (green) in mouse Hepa1-6 tumor sections (representative of *n* = 3). **B** Surface staining and analysis of CD146 on tumor macrophages from Hepa1-6 tumors (*n* = 5). **C** Immunostaining of CD68 in human liver samples (representative of *n* = 3). **D** Immunostaining (DAPI, nuclear staining) of CD146 (red) and CD68 (green) in human HCC tumor sections (representative of *n* = 3). **E** Surface staining of CD45, CD3, CD11b, CD14, HLA-DR, CD163 and CD146 in immune cells from HCC patient tumors. Percentages of the indicated cell populations in CD45^+^ cells (**F**), TAMs in CD11b^+^ cells or CD146^+^ cells (**G**) in TAMs from liver samples (*n* = 5). **H** Indicated cell population counts per tissue by weight (*n* = 5). Each symbol (**B**, **F**–**H**) represents an individual sample. A two-tailed *t* test (**B**) or one-way ANOVA (**F**–**H**) was performed. Data are shown as the mean ± SEM. *P < 0.05, **P < 0.01, ***P < 0.001

To unambiguously elucidate the expression of CD146 in TAMs within tumors, we first investigated the CD146 expression in TAMs from mouse and human tumor tissue. In liver cancer model mice, CD146 was expressed on a subset of CD11b^+^ myeloid cells (Fig. 1A). Furthermore, flow cytometry (FCM) analysis showed that it was expressed on approximately 40% of macrophages (CD11b^+^F4/80^+^; Fig. 1B). To investigate the expression and distribution of CD146^+^ macrophages in human liver cancer samples, we collected liver cancer tissue, tumor-adjacent tissue and relatively normal marginal tissue from five patients undergoing liver cancer surgery. Immunohistochemical (IHC) staining results indicated that CD68^+^ macrophages were evenly distributed in normal and tumor-adjacent tissue, while in tumor tissue, they were mainly enriched around the stroma (Fig. 1C). CD146 was expressed on a subset of CD68^+^ macrophages in the tumor margins between tumor tissue and tumor-adjacent tissue (Fig. 1D), which was confirmed by FCM. As shown in Fig. 1E, CD146 was expressed in a subset of CD45^+^ CD11b^+^ CD3^−^ HLADR^+^ CD14^+^ CD163^+^ TAMs. In the three types of tissue, CD3^+^ T cells accounted for approximately 60% of all leukocytes, while the proportion of CD11b^+^ myeloid cells was approximately 20–35% depending on individual differences in patients, with relatively higher levels in tumor tissue (Fig. 1F). Although the proportion of macrophages overall showed little difference in CD11b^+^ cells among tissue types, the proportion of CD146^+^ macrophages was significantly higher in tumor-adjacent tissue than in normal or tumor tissue (Fig. 1G). Our count showed that the proportions of CD11b^+^ and CD3^+^ cells per gram were reduced in tumor tissue. While the overall TAM count was similar across all three tissue types, there were more CD146^+^ TAMs in both normal and tumor-adjacent tissues than in tumor tissue, with tumor-adjacent tissue having the highest count (Fig. 1H). Altogether, these data implied that CD146^+^ macrophages are excluded from the intratumor region.

### CD146 expression on macrophages was controlled by STAT3 signaling in the tumor microenvironment

**Fig. 2:**
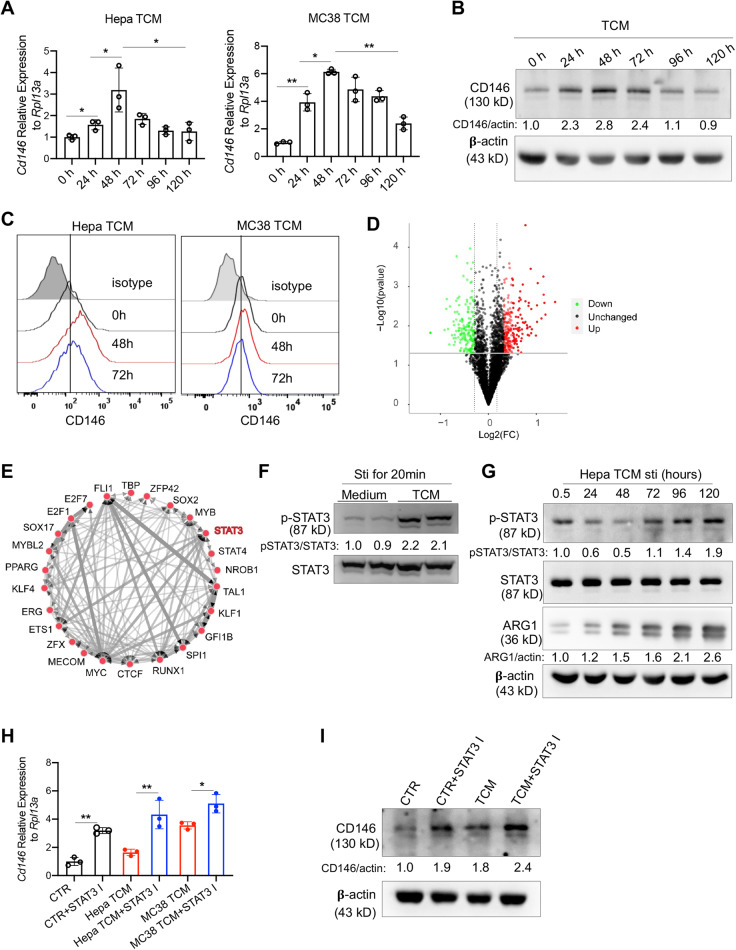
Expression of CD146 in macrophages was controlled by the TME. **A** *Cd146* gene expression in BMDMs stimulated with TCM for the indicated times. **B** Western blotting (WB) for CD146 protein in BMDMs stimulated as mentioned above. β-Actin served as the loading control. **C** Surface staining of CD146 on BMDMs stimulated as mentioned above. **D** Volcano plot comparing the expression levels of proteins from macrophages treated with or without Hepa1-6 TCM. **E** TFEA of differentially expressed proteins after Hepa-TCM treatment via a web-based TFEA tool, ChIP-X Enrichment Analysis (ChEA3) v3. **F** WB for p-STAT3 and STAT3 of BMDMs stimulated with or without TCM for 20 min. **G** WB for p-STAT3, STAT3 and ARG1 proteins in BMDMs stimulated with TCM for the indicated times. ARG1 served as the M2 marker. β-Actin served as the loading control. **H** *Cd146* gene expression in BMDMs stimulated with TCM, supplemented or not supplemented with *STAT3* inhibitor for 72 h. **I** WB for CD146 protein in BMDMs stimulated with TCM for the indicated time with or without the STAT3 inhibitor. β-Actin served as the loading control. Each symbol (**A**, **H**) represents an individual experiment. One-way ANOVA (**A**, **H**) was performed. Data are shown as the mean ± SEM. *P < 0.05, **P < 0.01

The reduced number of CD146^+^ macrophages in intratumor tissue had several potential causes, such as apoptosis, lower migration capability or loss of CD146 expression on macrophages. The results showed that rate of apoptosis was similar between CD146^+^ and CD146-deleted macrophages (Fig. S1A). Moreover, CD146 deletion did not significantly affect the number and proliferation of macrophages under short (24–48 h) and long (72–96 h) tumor cell-conditioned medium (TCM) stimulation (Fig. S1B, C). Further experiments showed that CD146 was upregulated under the stimulation of TCM initially (24–48 h) but was downregulated under longer stimulation (72–96 h; Fig. 2A–C). In addition, the M1-like markers *iNos, Tnfa, Il1b, and Il6* were upregulated with short-term stimulation but downregulated by long-term stimulation; the levels of the M2-like markers *Arg1, Ym1, Il10, and Mgl1* were highest after a prolonged stimulation period (Fig. S1D–E), suggesting the switch from a proinflammatory phenotype to an anti-inflammatory phenotype in these macrophages in the tumor microenvironment. Moreover, long-term TCM stimulation (72 h) promoted macrophage migration (Fig. S1F). These data suggested that continuous TCM stimulation reduced CD146^+^ macrophages in intratumor tissue at least partially by downregulating CD146 expression.

To determine which signaling pathway controls CD146 expression, we performed a comparative proteomics analysis using TCM-stimulated or non–TCM-stimulated macrophages (Fig. S1G, H). The data showed that under long TCM stimulation, 128 proteins were upregulated and 82 were downregulated (≥1.25-fold or ≤0.7-fold, Fig. 2D, Table S1). Based on these proteins, we performed transcription factor enrichment analysis (TFEA). As shown in Fig. 2E, 25 transcription factors (TFs) were found to regulate the differential protein expression. Since *STAT3* is an important TF for M2-like polarization [19] and our previous study showed that *STAT3* signaling promotes M2-like polarization with CD146 downregulation [17], we focused on *STAT3* activation. Indeed, we detected strong *STAT3* activation in TCM-stimulated macrophages by Western blotting (WB; Fig. 2F). In addition, the activation of *STAT3* increased with prolonged TCM stimulation (Fig. 2G). TF knockdown analysis (http://www.licpathway.net/KnockTF/analysis) indicated that CD146 was negatively regulated by *STAT3* **(**Fig. S1I**)**. By using a *STAT3* inhibitor, we found that CD146 expression was significantly upregulated in macrophages (Fig. 2H–I). Overall, these data suggested that macrophage CD146 expression was controlled at least partially by TCM-induced *STAT3* signaling and confirmed that CD146^+^ macrophages were reshaped in the TME, suggesting that the loss of CD146 expression in macrophages affected their role in tumor development.

### CD146 expression on macrophages inhibited tumor development

**Fig. 3:**
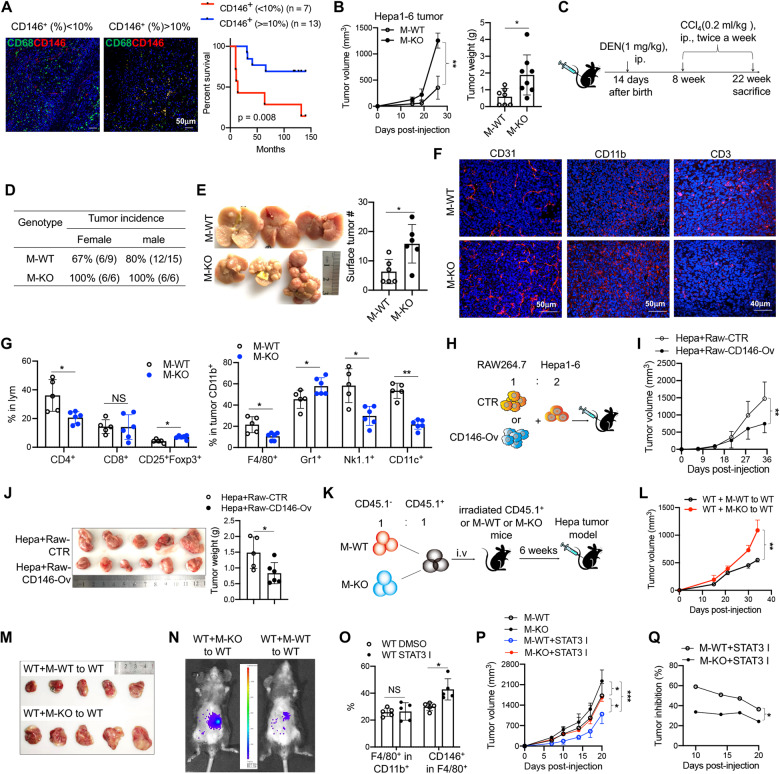
CD146 expression in macrophages inhibited tumor development. **A** Kaplan–Meier curves showing the overall survival analyses for HCC patients with different percentages of CD146^+^ macrophages. **B** Hepa1-6 tumor development and weight in M-WT and M-KO mice (*n* = 8). **C** Schematic representation of the DEN-induced HCC mouse model. **D** DEN-induced HCC tumor incidence in M-WT and M-KO mice. **E** Representative DEN-induced HCC tumors. Right panel, surface tumor numbers in livers from M-WT and M-KO mice (*n* = 6). **F** Immunofluorescence (IF) staining of CD31, CD11b or CD3 in Hepa1-6 tumors. **G** FACS analysis of F4/80^+^, CD11b^+^ Gr-1^+^ (MDSC), CD4^+^ CD25^+^ Foxp3^+^, CD11b^+^ NK1.1^+^ and CD11b^+^ CD11c^+^ subpopulations in Hepa1-6 tumors from M-WT (*n* = 5) or M-KO mice (*n* = 6). **H** Schematic representation of the coinjection tumor model. **I**, **J** Coinjection tumor model development (**I**) and tumor weight (**J**) (*n* = 5). **K** Schematic representation of the chimeric bone marrow-transferred tumor implantation model. **L**, **M** Hepa1-6 tumor development (**L**) and weight (**M**) in chimeric bone marrow-transferred mice. **N** Representative imaging of orthotopic liver transplantation tumors with Hepa1-6 cells (*n* = 3). **O** FACS analysis of F4/80^+^ or CD146^+^ F4/80^+^ cell percentages in *STAT3* inhibitor-treated mice and control mice (*n* = 5). **P** Hepa1-6 tumor development in groups as indicated (*n* = 5). **Q** Tumor inhibition of the *STAT3* inhibitor in M-WT or M-KO mice (*n* = 5). Each symbol (**B**, **E**, **G**, **J**, **O**) represents an individual mouse. A two-tailed *t* test (**B**, **E**, **J**), one-way ANOVA followed by Bonferroni’s correction (**G**, **O**), two-way ANOVA with multiple-comparison test (**B**, **I**, **L**) or two-way repeated-measures (RM) ANOVA with Tukey’s test (**P**, **Q**) was performed. Data are shown as the mean ± SEM. NS not significant. *P < 0.05, **P < 0.01, ***P < 0.001

To clarify the role of CD146^+^ macrophages in tumor development, we first analyzed the relationship between the percentage of CD146^+^ macrophages and HCC patient survival. As shown in Fig. 3A, the higher the percentage of CD146^+^ macrophages in the tumor, the longer the patient survival, suggesting that CD146^+^ macrophages may have antitumor activity. To investigate this notion, we constructed macrophage CD146 conditional knockout (M-KO) mice (Fig. S2A, B) and established a subcutaneous transplant tumor mouse model using a mouse liver cancer cell line (Hepa1-6) or colorectal cancer cell line (MC38) in these mice and their control wild-type littermates (M-WT). Tumor growth was measured every 3 days. The results showed that both types of tumors grew faster in M-KO mice than in M-WT mice (Fig. 3B and Fig. S2C, D). In addition, in a diethylnitrosamine (DEN)-induced in situ model of hepatocellular carcinoma (HCC), compared to control mice, mice with macrophage CD146 deficiency had a higher tumor incidence and more surface tumors (Fig. 3C–E). Consistent with these data, tumors in M-KO mice had more blood vessels, increased infiltration of myeloid cells and fewer CD3^+^ T cells (Fig. 3F), suggesting an immunosuppressive environment in these mice. FCM analysis confirmed these results. As shown in Fig. 3G, we observed fewer T cells, dendritic cells (DCs) and natural killer (NK) cells but more CD11b^+^Gr-1^+^ MDSCs and regulatory T cells (Tregs) in tumors from M-KO mice than in those from M-WT mice. In DEN-induced liver cancer, although we observed no significant difference in the percentages of DCs and NK cells, more CD11b^+^Gr-1^+^ MDSCs (including M-MDSCs and G-MDSCs) and Tregs were observed in the TME (Fig. S2E, F), confirming the immunosuppressive environment in M-KO mice. All of these data suggested that CD146^+^ macrophages inhibited tumor development.

To demonstrate whether increasing the number of CD146^+^ macrophages would improve antitumor activity, we established a model in which tumor cells (Hepa1-6 or MC38) were mixed with a 0.5-fold number of Raw264.7 cells transfected with empty plasmid (CTR) or *Cd146*-plasmid (CD146-Ov) (Fig. S2G) and then subcutaneously injected into WT mice (Fig. 3H). As shown in Fig. 3I–J and Fig. S2H–I, tumor cells mixed with CD146-overexpressing Raw264.7 cells significantly inhibited tumor growth. These data suggested that CD146 augmented the antitumor activity of macrophages.

To confirm the cell-intrinsic role of CD146 in the antitumor activity of macrophages, we used a bone marrow chimeric mouse model. Briefly, bone marrow at a 1:1 ratio from CD45.1^+^ (WT) donors and CD45.2^+^CD45.1^–^ donors that were CD146 M-WT (mixed WT-WT) or CD146 M-KO (mixed KO-WT) was transferred into lethally irradiated CD45.1^+^ host mice or M-WT or M-KO host mice (Fig. 3K). After 6 weeks, the chimeric mice were used to establish subcutaneous transplanted tumor models or orthotopic liver transplantation tumor models with Hepa1-6 cells. As shown in Fig. 3L, M, in the Hepa1-6 subcutaneous transplanted tumor model, tumors grew larger in M-KO HSC-transferred mice than in M-WT HSC-transferred mice. In addition, in the orthotopic liver transplantation tumor model, M-KO HSC-transferred mice also developed larger tumors than M-WT HSC-transferred mice (Fig. 3N), suggesting the intrinsic antitumor activity of bone marrow-derived macrophages.

We showed that a *STAT3* inhibitor upregulated CD146 expression in macrophages. *STAT3* inhibitors have been evaluated in clinical trials for advanced tumor treatment [7]. In this study, we found that tumor-bearing WT mice treated with a *STAT3* inhibitor showed a reduction in the proportion of CD11b^+^Gr-1^+^ MDSCs (including M-MDSCs and G-MDSCs) and an increase in the proportion of CD11b^+^CD11c^+^ and CD3^+^ cells (Fig. S2J–K). Interestingly, although they had a similar proportion of total tumor F4/80^+^ macrophages, mice treated with the *STAT3* inhibitor exhibited an increase in in the proportion of tumor CD146^+^ macrophages compared with that in control mice (Fig. 3O). To test whether the antitumor activity of the *STAT3* inhibitor depended on CD146 expression on macrophages, we then treated tumor-bearing M-WT or KO mice with a *STAT3* inhibitor. As shown in Fig. 3P–Q, the *STAT3* inhibitor inhibited tumor growth by approximately 50% in M-WT mice and by approximately 30% in M-KO mice. Overall, these data suggested that tumor inhibition by *STAT3* inhibitors was at least partially dependent on CD146 expression in macrophages.

### The antitumor effect of CD146^+^ macrophages was partially dependent on T cells

**Fig. 4:**
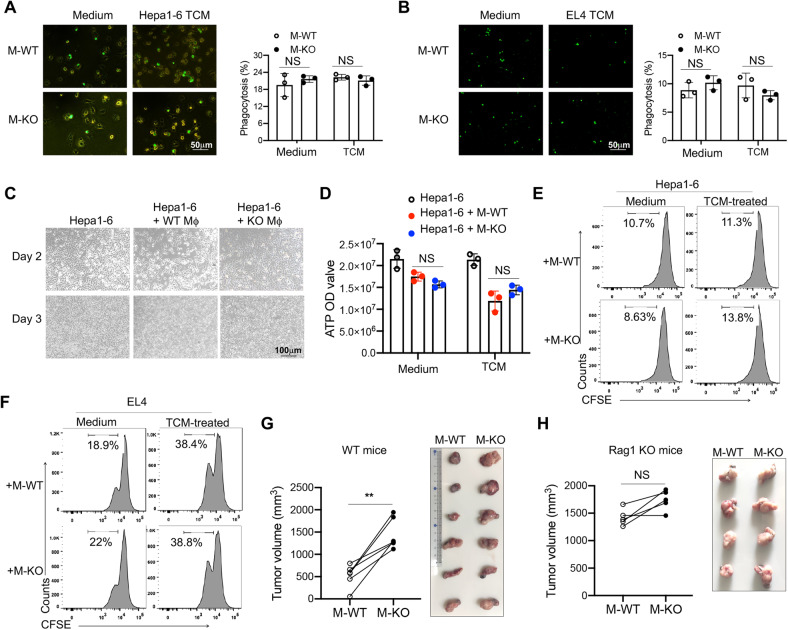
The antitumor effect of CD146^**+**^ macrophages mainly depended on T cells. **A**, **B** In vitro macrophage phagocytosis of Hepa1-6 (**A**) and EL4 (**B**) tumor cells (*n* = 3). **C** Representative image of Hepa1-6 tumor cells cocultured with or without CD146 WT or KO macrophages for the indicated number of days. **D** Determination of adenosine triphosphate (ATP) levels in Hepa1-6 cells cocultured for 48 h with TCM-pretreated macrophages (*n* = 3). **E**, **F** Proliferation assay for Hepa1-6 (**E**) or EL4 (**F**) cells cocultured for 24 h with medium or with TCM-pretreated macrophages. **G**, **H** MC38 tumor growth and weight in a model of subcutaneous coinjection of Raw264.7-CD146 cells into WT (**G**) (*n* = 6) or Rag1 KO mice (**H**) (*n* = 4). Each symbol represents an experiment (**A**, **B**, **D**) or an individual mouse (**G**, **H**). One-way ANOVA followed by Bonferroni’s correction (**A**, **B**, **D**) and a two-tailed *t* test (paired for **G**, **H**) were performed. Data are shown as the mean ± SEM. NS not significant. *P < 0.05, **P < 0.01

The role of macrophages in tumor growth depends on several mechanisms, such as regulating tumor angiogenesis, modulating the functions of other immune cells or directly acting on tumor cells (i.e., phagocytosis or promotion of tumor cell proliferation) [6, 20, 21]. To clarify the mechanism by which CD146-KO macrophages promoted tumor development, we first measured phagocytosis of M-WT and M-KO macrophages. After coculturing Hepa1-6 or EL4 cells with macrophages, we observed no significant difference in phagocytosis between M-WT and M-KO macrophages at either 2 h (Fig. 4A, B) or 48–72 h (Fig. 4C, D). In addition, we observed no difference in tumor proliferation between M-WT and M-KO macrophages (Fig. 4E, F). These data suggested that CD146 was not directly involved in the function of macrophages in tumor phagocytosis or proliferation.

Since the eradication of tumor cells mainly depends on T cells, we further determined whether macrophage CD146–mediated inhibition of tumor growth was dependent on T cells. After establishing a model in which we subcutaneously injected tumor cells mixed with a two-fold number of CD146 WT or KO macrophages into both flanks of WT and T-cell-deficient mice (Rag1 knockout), we found that tumor cells mixed with CD146-KO macrophages developed faster than those mixed with CD146 WT macrophages in WT mice (Fig. 4G). However, the difference in tumor growth was not significant in T-cell–deficient mice (Fig. 4H), indicating that CD146^+^ macrophage-mediated tumor suppression mainly depended on the presence of T cells.

### CD146 deletion on macrophages promoted the recruitment of CD11b^+^Gr-1^+^ MDSCs

**Fig. 5:**
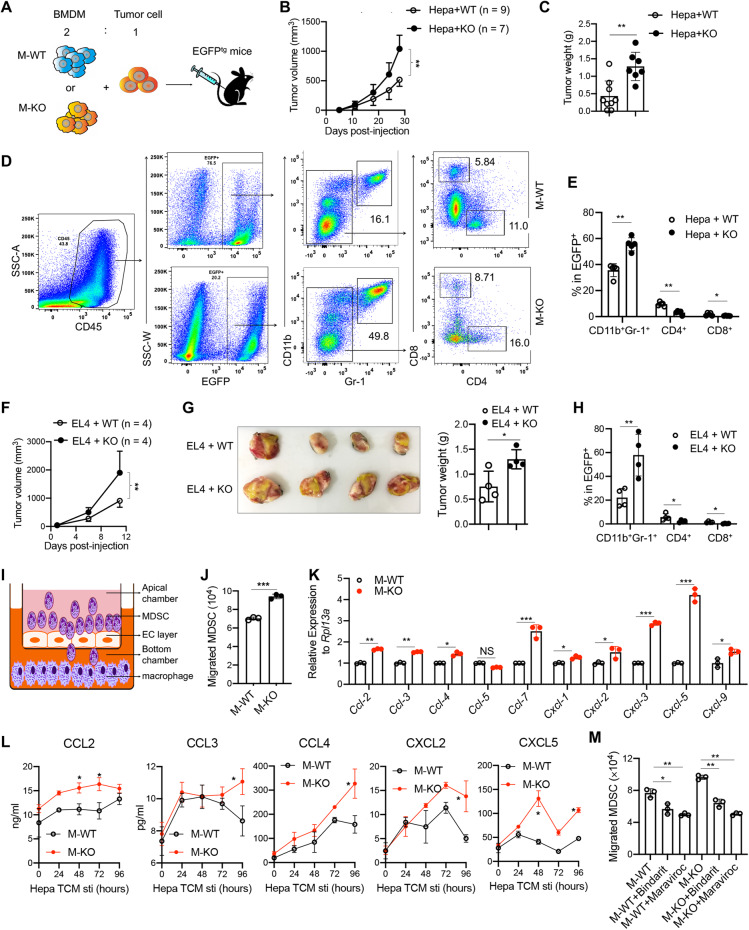
CD146 deletion in macrophages promoted the recruitment of MDSCs. **A** Schematic representation of the coinjection tumor model. **B**, **C** Hepa1-6 tumor development (**B**) and tumor weight (**C**) in the subcutaneous coinjection model in EGFP^tg^ mice. **D** FACS analysis of infiltrating MDSCs and CD4^+^ or CD8^+^ T cells in tumors from the coinjection model. **E** Percentages of MDSCs and T cells in the EGFP^+^ cell population from Hepa1-6 tumors. **F**, **G **EL4 tumor development (**F**) and weight (**G**) in the subcutaneous coinjection tumor model in EGFP^tg^ mice (*n* = 4). **H** Percentages of MDSCs and T cells in the EGFP^+^ cell population from EL4 tumors. **I** Schematic diagram of the in vitro MDSC transmigration assay. **J** MDSC migration induced by macrophage chemotaxis (*n* = 3). **K** Real-time PCR analysis of chemokine genes in M-WT and M-KO mice (*n* = 3). **L** ELISA for CCL2, CCL3, CCL4 and CXCL2 and CXCL5 in the supernatant of macrophages stimulated with TCM for the indicated times. **M** MDSC migration induced by chemotaxis of macrophages in the presence of Bindarit or Maraviroc (*n* = 3). Each symbol represents an individual mouse (**C**, **E**, **G**, **H**) or an experiment (**J**, **K**, **M**). Two-way ANOVA with multiple-comparison test (**B**, **F**, **L**), two-tailed *t* test (**C**, **G**, **J**) or one-way ANOVA followed by Bonferroni’s correction (**H**, **K**, **M**) was performed. Data are shown as the mean ± SEM. *P < 0.05, **P < 0.01, ***P < 0.001

Due to the immunosuppressive TME in M-KO mice and the importance of MDSCs as immunosuppressive cells that play roles involving T cells and other tumor stromal cells, we hypothesized that M-KO macrophages were associated with the recruitment of MDSCs. To test this supposition, we established a tumor model by mixing Hepa1-6 cells with M-WT or M-KO macrophages and subcutaneously coinjecting them into enhanced green fluorescent protein (EGFP)-transgenic mice (Fig. 5A). We found that Hepa1-6 tumor cells mixed with M-KO macrophages grew faster than those mixed with M-WT macrophages (Fig. 5B, C), a finding consistent with the aforementioned data. By isolating tumor-infiltrated EGFP^+^ leukocytes, we found that in tumors mixed with M-KO macrophages, CD11b^+^ Gr-1^+^ MDSCs (approximately 50%) were enriched, especially Ly6G^+^ G-MDSCs, while the infiltration of CD4^+^ and CD8^+^ T cells was lower (Fig. 5D, E, Fig. S3A). These data were confirmed in the EL4 tumor model, which demonstrated substantial MDSC recruitment to the tumor microenvironment (Fig. 5F–H). These data suggested that M-KO macrophages promoted tumor development mainly by recruiting MDSCs to the TME.

To confirm the role of CD146-KO macrophages in MDSC recruitment, we performed an in vitro transmigration assay (Fig. 5I). CD11b^+^Gr-1^+^ MDSCs were isolated from EL4 tumor-bearing mice and then seeded onto the endothelial layer of the apical chamber. TCM-treated macrophages were cultured in the bottom chamber. The data showed that more MDSCs were recruited to the bottom chamber containing M-KO macrophages (Fig. 5J), suggesting that M-KO macrophages promoted MDSC recruitment, most likely as a result of the cytokines or chemokines secreted from M-KO macrophages.

To elucidate which cytokine or chemokine promoted MDSC recruitment in M-KO mice, we first assessed the protein levels of cytokines or chemokines in M-WT and M-KO mice from our DEN-induced liver cancer model. We measured serum levels of 36 cytokines and chemokines using Luminex multiplex assays (R&D Systems, Inc., Minneapolis, MN, USA). As shown in Fig. S3B, the levels of 16 of the 36 factors were higher in M-KO mice than in M-WT mice including tumor necrosis factor alpha (TNF-α); interferon gamma (IFN-γ); IL-1β, −6, −10, −12p70, −22, −18, and −28; chemokine (C-C motif) ligands 2, 3, 4 and 7 (CCL2, CCL3, CCL4, CCL7); and C-X-C motif chemokines 1, 2 and 5 (CXCL1, CXCL2, CXCL5). Since chemokines, such as CCL2 and CCL3 and CXCL2 and CXCL5, are reported to be associated with MDSC recruitment [22], we further measured chemokine mRNA levels in M-WT and M-KO macrophages using real-time PCR. As shown in Fig. 5K, the expression of *Ccl2, Ccl3, Ccl4, Ccl7, Cxcl1, Cxcl2, Cxcl3* and *Cxcl5* was significantly upregulated in M-KO macrophages under TCM stimulation. In addition, we measured the *CCL2, CCL3, CCL4, CXCL2*, and *CXCL5* protein levels secreted by M-WT and M-KO cells. As shown in Fig. 5L, M-KO mice expressed higher levels of these chemokines. These data suggest that CD146-KO macrophages promote MDSC recruitment mainly by producing these chemokines. To confirm this conclusion, the chemokine inhibitor bindarit was added to the bottom chamber, or the CCR5 inhibitor maraviroc was added to the upper chamber. The data show that these inhibitors significantly blocked the M-KO-induced recruitment of MDSCs (Fig. 5M), suggesting that the promotion of MDSC recruitment by M-KOs was at least partially dependent on chemokine expression.

### CD146-deleted macrophages promoted the recruitment of MDSCs via *JNK* signaling

**Fig. 6:**
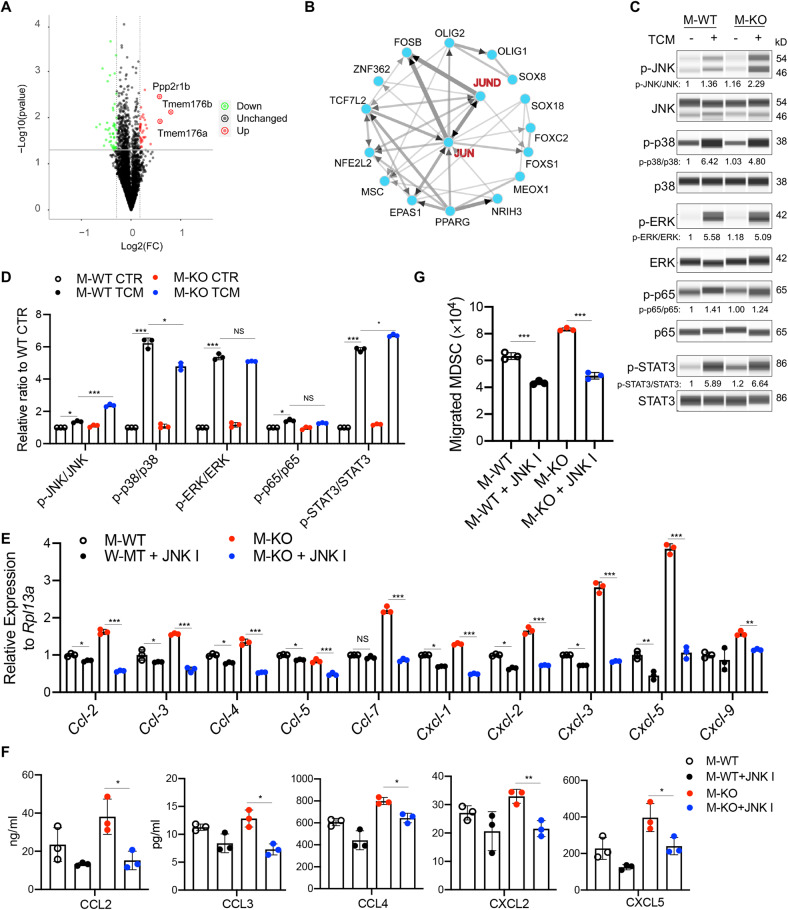
CD146-deleted macrophages promoted the activation of *JNK* signaling. **A** Volcano plot comparing the expression levels of proteins from Hepa-TCM–treated M-WT and M-KO macrophages. **B** TFEA of differentially expressed proteins from Hepa-TCM–treated M-KO macrophages using ChEA3 v3. **C** WB analysis of p-*JNK*, p-*p38*, p-*ERK*, p-*p65* and p-*STAT3* levels in macrophages. Total *JNK*, *p38*, *Erk*, *p65* and *STAT3* proteins were loaded as controls. **D** Quantitative analysis of p-*JNK*/*JNK*, p-*p38*/*p38*, p-*ERK*/*ERK*, p-*p65*/*p65* and p-*STAT3*/*STAT3* ratios in macrophages under TCM stimulation (*n* = 3). **E** Real-time PCR analysis of chemokine genes in M-WT and M-KO mice treated or not treated with *JNK* inhibitor (*n* = 3). **F** ELISA for CCL2, CCL3, CCL4 and CXCL2 and CXCL5 in the supernatant of macrophages stimulated with TCM supplemented  with or without JNK inhibitor (*n* = 3). **G** MDSC migration induced by *JNK* inhibitor-treated macrophages (*n* = 3). Each symbol represents an individual experiment (**D**–**G**). One-way ANOVA followed by Bonferroni’s correction (**D**–**G**) was performed. Data are shown as the mean ± SEM. NS not significant. *P < 0.05, **P < 0.01, ***P < 0.001

To explore the mechanism by which CD146 regulates cytokine expression, we performed a proteomics analysis. As shown in Fig. 6A, we identified 26 upregulated and 21 downregulated proteins in M-KO macrophages compared to M-WT macrophages (Table S2). To assess how these proteins were regulated, we analyzed the TFs that control most regulated proteins. *Jun* was the most influential of these (Fig. 6B), which suggested that *JNK* signaling was considerably altered in M-KO macrophages. To confirm this hypothesis, we stimulated macrophages with TCM and analyzed the activation of the signaling pathways. We observed that TCM stimulation activated *JNK*, p38, extracellular signal–regulated kinase (*ERK*), nuclear factor κ-light-chain-enhancer of activated B cells (*NF-κB*) and *STAT3* signaling. However, when CD146 was deleted, the most influential signaling pathway was *JNK*, as shown by higher levels of phosphorylated *JNK* (p-*JNK*) in M-KO macrophages (Fig. 6C, D). Overall, these data suggested that in the TME, CD146 negatively controlled *JNK* signaling in macrophages.

To determine whether M-KO macrophages promoted MDSC recruitment via *JNK* signaling, we first measured the expression levels of chemokine genes and proteins in M-WT and M-KO macrophages under TCM stimulation in the presence of a *JNK* inhibitor. As shown in Fig. 6E, F, while TCM stimulation induced higher levels of cytokines in M-KO macrophages, the *JNK* inhibitor markedly reduced these levels, suggesting that M-KO macrophages expressed cytokines mainly via *JNK* signaling. Consistent with this finding, the *JNK* inhibitor blocked the recruitment of MDSCs (Fig. 6G). These data collectively suggested that M-KO macrophages promoted MDSC recruitment at least partially by activating *JNK* signaling.

### CD146 positively regulated NLRP3 inflammasome activation by *JNK* signaling

**Fig. 7:**
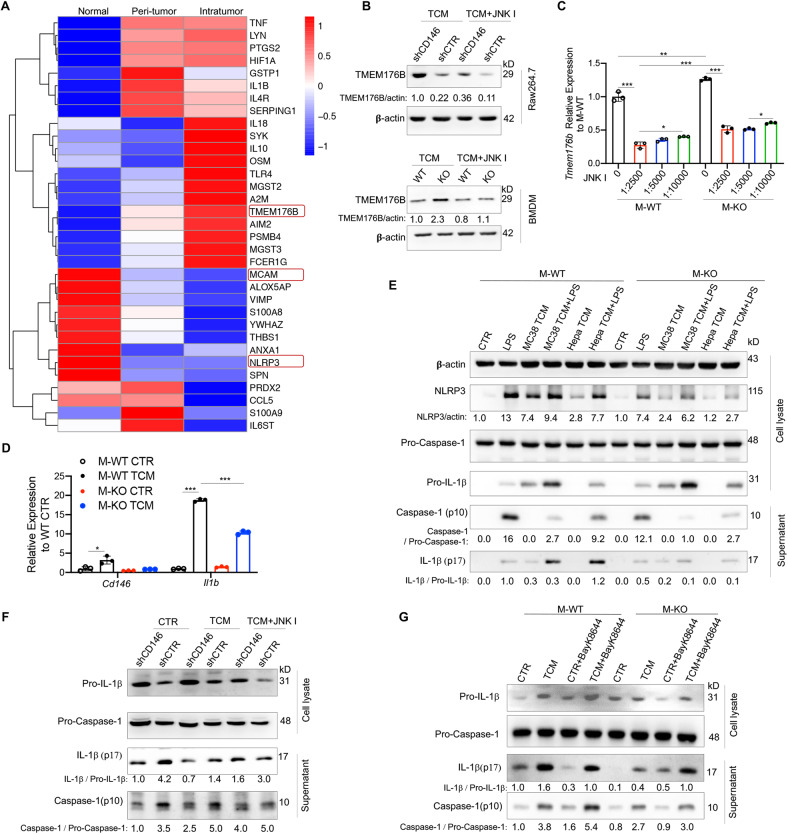
CD146 negatively regulated the expression of TMEM176B. **A** Heatmap of the relative expression of CD146 (MCAM) and inflammatory response-associated DE genes among macrophage subsets of N, PT, and T samples. **B** WB analysis of TMEM176B in RAW264.7 cells or BMDMs. Actin served as a loading control. **C** Real-time PCR analysis of the *Tmem176b* gene in M-WT and M-KO mice treated or not treated with a JNK inhibitor (JNK I, 25 mM) (*n* = 3). **D** Real-time PCR analysis of *Cd146* and *Il1b* genes in M-WT and M-KO mice treated with TCM for 24 h (*n* = 3). **E** WB analysis of actin, NLRP3, pro-IL-1β, pro-Caspase-1, IL-1β and Caspase-1 in a cell lysate or supernatant from M-WT or M-KO macrophages stimulated with LPS (10 ng/ml) and/or TCM for 12 h, followed by 5 mM ATP for 1.5 h. **F** WB analysis of pro-IL-1β, pro-Caspase-1, IL-1β and Caspase-1 in a cell lysate or supernatant from CD146-knockdown Raw264.7 cells stimulated with TCM for 12 h, followed by 5 mM ATP for 1.5 h. **G** WB analysis of pro-IL-1β, pro-Caspase-1, IL-1β and Caspase-1 in the cell lysate or supernatant from BMDMs stimulated with TCM for 12 h in the presence of Bayk8644. Each symbol represents an individual experiment (**C**, **D**). One-way ANOVA followed by Bonferroni’s correction (**C**, **D**) was performed. Data are shown as the mean ± SEM. *P < 0.05, **P < 0.01, ***P < 0.001

We next explored how to enhance the antitumor activity of TAMs. Using proteomics analysis, we found that TMEM176B was markedly upregulated in M-KO macrophages (Fig. 6A). By isolating CD11b^+^CD14^+^CD163^+^ macrophages from normal, peritumor, and intratumor tissues and performing RNA sequencing, we found that macrophage *TMEM176B* expression levels were lower in normal tissue but higher in tumor tissue, inversely correlating with the expression of CD146 (Fig. 7A). A recent paper reported that myeloid TMEM176B is a potential modulator of tumor immunotherapy [18]. Therefore, we speculated that TMEM176B was negatively regulated by CD146 and controlled tumor development. As shown in Fig. 7B, under TCM stimulation, TMEM176B expression was significantly upregulated in M-KO cells or CD146-KO Raw264.7 cells compared with M-WT cells. In addition, TMEM176B expression was partially controlled by *JNK* signaling. Based on the aforementioned data that CD146 negatively regulates *JNK* activation, we hypothesized that CD146 negatively regulates TMEM176B expression via *JNK* signaling. We supported this hypothesis by assessing mRNA levels (Fig. 7C).

TMEM176B has also been reported to inhibit Caspase-1 activation and IL-1β maturation, both of which are markers for inflammasome activation [23]. Based on the negative relationship between CD146 and TMEM176B, we speculated that CD146 was involved in inflammasome activation. Indeed, *Il1b* expression levels were significantly lower in CD146-deleted macrophages than in M-WT cells under TCM stimulation for 24 h (Fig. 7D). To verify this finding, we measured Caspase-1 activation and IL-1β maturation in CD146 WT and KO macrophages. As shown in Fig. 7E, TCM stimulation for 12 h promoted the activation of Caspase-1 and maturation of IL-1β in both CD146 WT and KO macrophages. However, CD146 WT macrophages showed higher activated Caspase-1 levels and mature IL-1β levels than CD146-KO macrophages, as observed by the proportions of Caspase-1/pro-caspase-1 and IL-1β/pro-IL-1β protein levels. These data suggested that CD146 positively regulates NLRP3 inflammasome activation. This finding was further confirmed with lipopolysaccharide (LPS)-induced inflammasome activation experiments in vitro and in vivo (Fig. 7E, Fig. S4). By using CD146-knockdown RAW264.7 cells, we confirmed the positive regulation of inflammasome activation by CD146. Interestingly, the addition of a *JNK* inhibitor increased both Caspase-1 activation and IL-1β maturation (Fig. 7F). BayK8644, a potent TMEM176B inhibitor, also significantly increased both Caspase-1 activation and IL-1β maturation in M-KO mice (Fig. 7G). These data suggested that CD146 is involved in inflammasome activation, functioning at least partially through TMEM176B.

### CD146 deletion-induced MDSC recruitment partially depends on TMEM176B

**Fig. 8:**
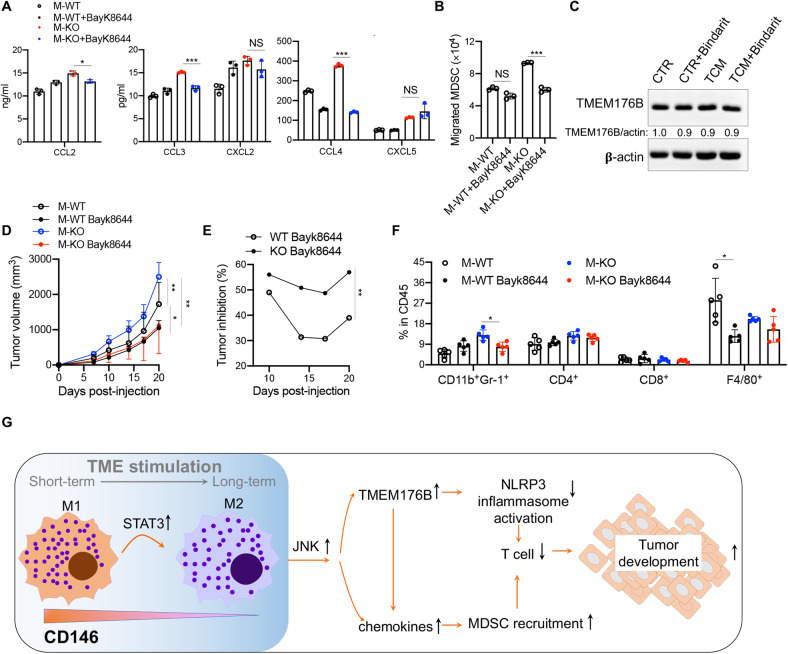
CD146 deletion-induced MDSC recruitment partially depends on TMEM176B. **A** ELISA for CCL2, CCL3, CCL4 and CXCL2 and CXCL5 using the supernatant from macrophages stimulated with TCM for 72 h in the presence of BayK8644. **B** MDSC migration induced by chemotaxis of macrophages in the presence of BayK8644 (*n* = 3). **C** WB analysis of TMEM176B in M-KO macrophages treated with bindarit. **D**, **E** Tumor growth (**D**) or inhibition (**E**) by BayK8644 in MC38 tumor-bearing M-WT or M-KO mice (*n* = 5). **F** FACS analysis of CD11b^+^Gr-1^+^, CD4^+^, CD8^+^ and F4/80^+^ cell populations in tumors from M-WT and M-KO mice treated or not treated with BayK8644 (*n* = 5). **G** Schematic diagram of the mechanism of CD146^+^ macrophages in tumor development. In the TME, during the short-term stimulation, macrophage upregulates the CD146 expression. With the long-term stimulation, the STAT3 activation in macrophage downregulates the CD146 expression, following by unleashing the activation of JNK, which promotes the expression of MDSC-recruitment associated chemokines as well as TMEM176B expression, the later inhibits the inflammasome activation. These factors block the T cell anti-tumor activity and then promote the tumor development. Each symbol represents an individual experiment (**A**, **B**) or mouse (**F**). One-way ANOVA followed by Bonferroni’s correction (**A**, **B**, **F**) or two-way ANOVA with multiple-comparison test (**D**, **E**) was performed. Data are shown as the mean ± SEM. *P < 0.05, **P < 0.01, ***P < 0.001

To determine whether the antitumor effect of CD146^+^ macrophages depends on TMEM176B, chemokines, or both, we performed additional experiments using ELISA to assess the chemokine levels after TMEM176B inhibition. The data showed that the inhibition of TMEM176B by BayK8644 in M-KO macrophages downregulated CCL2, -3 and -4 expression. However, the CXCL2 and CXCL5 levels were similar regardless of the presence of the TMEM176B inhibitor (Fig. 8A). In addition, the TMEM176B inhibitor Bayk8644 partially blocked M-KO-induced MDSC transmigration in vitro (Fig. 8B). These data suggest that TMEM176B may regulate the expression of some chemokines in M-KO macrophages. We also tested whether chemokine expression regulates TMEM176B expression. Treatment with the chemokine inhibitor bindarit revealed no significant changes in TMEM176B expression in M-KO macrophages (Fig. 8C). These data suggest that CD146 deletion in macrophages upregulates the expression of TMEM176B and some chemokines, such as CXCL2 and CXCL5, while TMEM176B expression also controls other chemokines, such as CCL2, CCL3 and CCL4, promoting MDSC migration.

To test whether CD146^+^ macrophages controlled tumor development through TMEM176B in vivo, we established a tumor model in M-WT and M-KO mice, followed by treatment with BayK8644. The data showed that in M-WT mice, BayK8644 treatment inhibited tumor growth by approximately 35%, while in M-KO mice, the inhibition rate was approximately 55% (Fig. 8D, E). FCM analysis showed less infiltration of MDSCs in tumors from BayK8644-treated M-KO mice, accompanied by a slight increase in the number of T cells **(**Fig. 8F). These data suggested that tumor promotion in M-KO mice at least partially depends on TMEM176B expression.

Overall, the downregulation of CD146 in TAMs promotes the activation of JNK signaling, thereby upregulating the expression of TMEM176B and MDSC recruitment-related chemokines, which in turn accelerate tumor development (Fig. 8G).

### Combination TMEM176B inhibitor + anti-CD146 therapy enhanced antitumor immunity

**Fig. 9:**
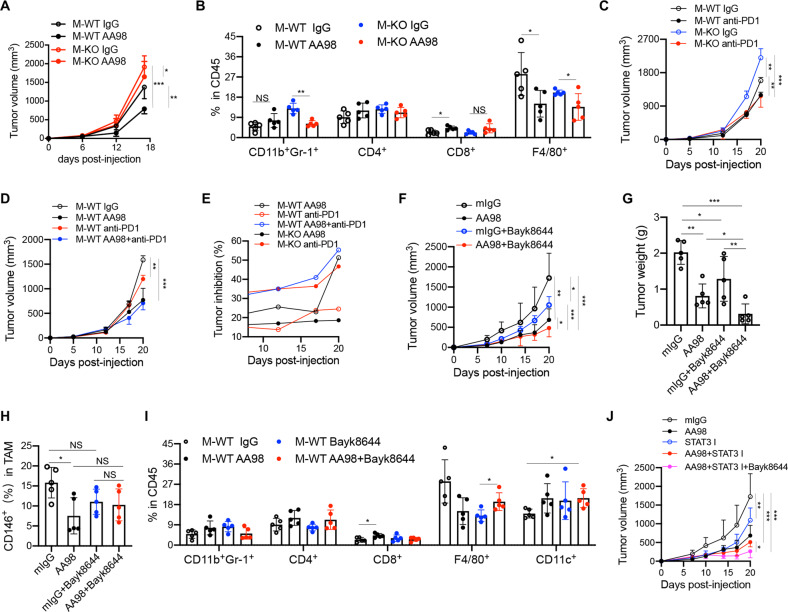
Combination TMEM176B inhibitor + anti-CD146 therapy enhanced antitumor immunity. **A** MC38 tumor growth in M-WT or M-KO mice treated with anti-CD146 AA98 (200 μg/mouse) 2×/week (*n* = 5). **B** FCM analysis of MDSC (CD11b^+^ Gr-1^+^), CD4^+^, CD8^+^ and CD11b^+^ F4/80^+^ percentages in total CD45^+^ leukocytes isolated from tumors (*n* = 5). **C**–**E** MC38 tumor growth and inhibition (**E**) in M-WT and M-KO mice treated with anti-PD-1 (**C**) and in WT mice treated with AA98 and/or anti-PD-1 (**D**) (*n* = 5). **F**, **G** MC38 tumor growth (**F**) and weight (**G**) in C57 mice treated with AA98 and/or BayK8644 (1 mg/kg) 2×/week (*n* = 5). **H**, **I** FCM analysis of CD146^+^ TAMs (**H**) and MDSCs, CD4^+^, CD8^+^ CD11b^+^F4/80^+^ and CD11b^+^CD11c^+^ percentages in total CD45^+^ leukocytes isolated from tumors (**I**) (*n* = 5). **J** MC38 tumor growth in C57 mice treated with AA98, *STAT3* inhibitor (STAT3 I) and/or BayK8644 2×/week (*n* = 5). Each symbol (**B**, **G**, **H,** **I**) represents an individual mouse. Two-way ANOVA with multiple-comparison test (**A**, **C**, **D**, **F**, **J**) or one-way ANOVA (**B**, **G**–**J**) was performed. Data are shown as the mean ± SEM. *P < 0.05, **P < 0.01, ***P < 0.001

The CD146 molecule is considered a marker for tumor angiogenesis and has been identified as a cancer therapeutic target [10, 24]. Our previous studies have shown that targeting CD146 with its functional antibody AA98 leads to 10–70% tumor inhibition depending on the cancer type [10, 25]. We then tested whether AA98’s effect would be reduced in tumor-bearing M-KO mice. As shown in Fig. 9A, AA98 inhibited MC38 tumor growth by approximately 50% in WT mice, but inhibition was <20% in M-KO mice, suggesting that blocking or deleting CD146 in macrophages disrupted the antitumor activity of AA98. AA98-treated M-WT mice showed fewer TAMs and more CD8^+^ T cells than monoclonal-antibody (mAb) IgG (mIgG)-treated M-WT mice, suggesting that AA98 treatment reshaped the tumor immune microenvironment (TIME). However, in the absence of CD146^+^ TAMs, although the MDSC count was reduced in AA98-treated mice, the number of CD8^+^ T cells was not significantly different from that in mIgG-treated mice (Fig. 9B).

An antibody for the immune checkpoint inhibitor (ICI) programmed cell death protein 1 (PD-1) has been approved for cancer therapy [26]. To test whether CD146^+^ macrophages enhanced the antitumor efficiency of anti–PD-1 antibodies, we treated MC38 tumor-bearing M-WT and M-KO mice with anti-PD-1 and/or anti-CD146 antibodies. As shown in Fig. 9C–E, tumor inhibition by anti–PD-1 treatment was approximately 20% in M-WT mice and approximately 50% in M-KO mice, while tumor growth in M-WT and M-KO mice did not show any difference after anti-PD-1 treatment, suggesting that the antitumor effect of ICIs is redundant with the effect of CD146^+^ macrophages. Since CD146 is also a biomarker of tumor angiogenesis, we hypothesized that blocking CD146 combined with ICIs would enhance antitumor efficiency. To confirm this, we treated WT mice with anti-CD146 AA98 and anti–PD-1 antibodies. As shown in Fig. 9D–E, AA98 treatment showed a better antitumor effect than anti-PD-1 treatment. Surprisingly, the combination of AA98 and anti-PD-1 did not augment the antitumor efficiency of AA98 alone. These data suggested that AA98 is a potential candidate agent for cancer therapy.

Since CD146^+^ macrophages play an important role in tumor prevention and because the deletion of CD146 on macrophages upregulates the expression of TMEM176B, to further improve the antitumor effect of AA98, we combined a TMEM176B inhibitor and AA98 in tumor therapy. As shown in Fig. 9F–G, combination AA98 + BayK8644 treatment inhibited tumor growth by 80%. Although the proportion of CD146^+^ TAMs, MDSCs, CD4^+^ cells and CD8^+^ cells showed no significant difference between the combination treatment group and any other group, the CD11c^+^ cell proportion was markedly increased in the combination treatment group (Fig. 9H–I). These data suggested that the combination of anti-CD146 and TMEM176B inhibitors could be a promising therapeutic strategy for tumor therapy. Interestingly, the *STAT3* inhibitor further enhanced the antitumor efficiency of AA98 alone or AA98 + anti-TMEM176B (Fig. 9J). Overall, these data suggested that modulating the function of TAMs augmented the efficiency of anti-CD146 antibodies in tumor therapy.

## Discussion

In this study, we identified a subtype of CD146^+^ macrophages as an antitumor population in the TME. CD146^+^ macrophages were located at the margins of tumors. With tumor growth, CD146 was downregulated via TME-induced *STAT3* activation. CD146 downregulation or deletion on macrophages activated the *JNK* pathway and then promoted the expression of MDSC recruitment-related cytokines or chemokines, thereby enhancing tumor immunosuppression. Impressively, deletion of CD146 upregulated the expression of TMEM176B and inhibited NLRP3 inflammasome activation. Using BayK8644 to inhibit TMEM176B markedly alleviated tumor growth in CD146 M-KO mice compared with M-WT mice. Combining the anti-CD146 antibody AA98 with BayK8644 enhanced antitumor efficiency over that of AA98 alone. Therefore, this study provided a new TAM-based strategy for cancer therapy.

The power of cancer immunotherapy has been revealed due to advances in basic, translational and clinical research [27, 28]. However, many cancer patients have exhibited resistance to such therapies [2]. Ongoing research indicates that resistance to ICIs or adoptive T-cell immunotherapies can be mediated by abundant tumor-associated myeloid cell infiltrates, including macrophages, monocytes and granulocytes. Macrophages are the most abundant immune cell component in the TME [29, 30]; transcriptome sequencing results of a variety of tumors showed that they account for approximately 30–50% of immune cell components in tumors [4, 31]. The most important characteristics of tumor macrophages are plasticity and heterogeneity, which make it difficult to fully clarify the functions and mechanisms of these macrophages. Over the past decade, evidence supporting complex properties and functions of macrophages in the context of the TME has accumulated [6, 21], but many questions remain regarding the relevant subpopulation and its location and functions in tumors. In this study, we found a new CD146^+^ macrophage that was mainly located in the margins of tumors and exerted antitumor function. This population of cells gradually lost its CD146 expression and then its antitumor function as the tumor grew. Decreasing CD146 expression levels in these macrophages facilitated MDSC recruitment to the TME by upregulating related cytokine or chemokine expression. In addition, we proved that this process was controlled by the *JNK* signaling pathway. TME-induced activation of *STAT3* might be the main cause of the loss of CD146 expression. STAT3 activation promotes the M2-like polarization of macrophages in the TME, which promotes immunosuppression [32]. Depletion of STAT3 in macrophages repolarized TAMs, inhibited tumor growth, and increased the infiltration of cytotoxic T cells in an autochthonous model of colorectal cancer [33]. In preclinical mouse models, a *STAT3* inhibitor could prevent tumor immunosuppression and synergize with ICIs to improve antitumor responses [34]. The *STAT3* inhibitor TTI-101 (Tvardi Therapeutics, Incorporated, USA) is now undergoing testing in a phase I clinical trial in patients with advanced cancers (clinicaltrials.gov ID NCT03195699) [7]. In our present study, a *STAT3* inhibitor was discovered to inhibit the downregulation of CD146 in macrophages, thereby maintaining macrophage antitumor activity. Indeed, our in vitro assay showed that CD146 expression levels were increased in TCM-stimulated macrophages in the presence of a *STAT3* inhibitor. Therefore, our study provides evidence for targeting *STAT3* in cancer therapy.

The M1-like polarization of macrophages is critical for tumor suppression [35]. The NLRP3 inflammasome has been reported to be involved in macrophage M1-like polarization [36]. The inflammasome is a high molecular weight protein complex formed in the cytosolic compartment as an inflammatory immune response to endogenous danger signals. NLRP3 inflammasome formation activates the inflammatory protease caspase-1, which triggers pyroptosis and cleaves the proinflammatory cytokines IL-1β and pro-IL-18 to generate their active forms [37]. Nod-like receptor protein 3 (NLRP3) is one of the most characterized inflammasome components that is mainly expressed in immune cells, such as macrophages. While the effect of NLRP3 activation on tumor development is controversial [38], the inflammasome-dependent activation of caspase-1 is reported to have an antitumor effect [18]. Our previous study showed that CD146 controls the proinflammatory polarization of macrophages under hyperlipid conditions, characterized by higher gene levels of *Il1b*, *Tnfa* and *Il6* [14], suggesting a correlation between CD146 and M1-like polarization. In the present study, we found that CD146 was positively associated with markers of inflammasome activation, such as activated caspase-1 and mature IL-1β. In addition, CD146-induced inflammasome activation was negatively controlled by *JNK* signaling. Higher JNK activation in CD146-deleted macrophages inhibited inflammasome activation. We also found that TMEM176B is a downstream target of JNK signaling in CD146-deleted macrophages. TMEM176B, also known as tolerance-related and induced (TORID), is a ubiquitously expressed protein [39] belonging to the CD20-like membrane-spanning 4A (MS4A) family and is highly expressed in monocytes, macrophages and CD11b^+^ DCs [39, 40]. It has also been identified as an immunoregulatory cation channel. A recent study identified TMEM176B as an innate immune checkpoint that curtails CD8^+^ T-cell–mediated immunity by repressing inflammasome activation. Targeting TMEM176B might influence antitumor effector mechanisms via Caspase-1/IL-1β activation [18]. Therefore, reduced CD146 expression levels may contribute to reduced inflammasome activation in TAMs. Manipulating inflammasome activation through CD146 augments the antitumor effect of macrophages.

We and other investigators have realized the critical role of CD146 in cancer therapy [10, 25]. CD146 expression is low or hardly detected in most healthy tissues. As tumors develop, CD146 is expressed in many tumor cells, neovascularized vessels and subtypes of immune cells [9]. CD146 in tumor cells or blood vessels has been proven to promote tumor development [10, 25], while in T cells, it plays a role in tumor suppression [15]. These opposing functions might explain why the efficiency of targeting CD146 varies among different cancer types. In addition, in this study, we found that CD146 on macrophages also exerted an antitumor effect. CD146 expression on macrophages has been observed in the context of hyperlipidemic conditions such as atherosclerosis and diet-induced obesity [14, 17]; in both conditions, CD146^+^ macrophages showed proinflammatory properties and promoted the development of unresolved chronic inflammation. In this study, in the TME, CD146^+^ macrophages exhibited M1-like properties and inhibited tumor development. However, the complexity of the TME induced CD146 downregulation and then inhibited the antitumor function of macrophages. The anti-CD146 antibody AA98 exerted a similar effect on macrophage function. Therefore, macrophages could partially neutralize the effect of targeting CD146 in tumor therapy. We also showed that TMEM176B is a candidate target molecule to compensate for the CD146 loss on macrophages. In this study, we clarified the relationship between CD146 and TMEM176B and combined them in tumor therapy, in which they showed great antitumor efficacy.

Altogether, we provided evidence that supports CD146^+^ macrophages as an antitumor population and elucidated how TAMs reshape the TME. We also provided a strategy for CD146-based antitumor treatment. Our study thus provides new insights into TAM-targeting strategies for cancer immunotherapy.

## Materials and methods

### Mice

Macrophage-specific CD146 knockout mice (CD146^M-Ko^, Lyz2^cre/+^ CD146^flox/flox^) and their control littermates (CD146^M-WT^, Lyz2^cre/+^ CD146^+/+^ or Lyz2^+/+^ CD146^flox/flox^) were generated as previously described [14]. We obtained female enhanced green fluorescent protein**–**transgenic mice (EGFP^tg^ mice) from the Nanjing Biomedical Research Institute of Nanjing University (Nanjing, China). Female C57BL/6J mice and nude mice were obtained from the Department of Laboratory Animal Science, Peking University Health Science Center (Beijing, China). Female Rag1 mice were kindly gifted by Prof. Pengyuan Yang (Institute of Biophysics, Chinese Academy of Sciences). All mice were maintained in a pathogen-free facility at the Animal Center of the Institute of Biophysics, Chinese Academy of Sciences (Beijing, China). All animal experiments were performed in compliance with the *Guidelines for the Care and Use of Laboratory Animals* and were approved by the Institutional Biomedical Research Ethics Committee of the Institute of Biophysics, Chinese Academy of Sciences (Permit No. SYXK2018-45).

### Human specimens

Four male and two female patients who were pathologically diagnosed with HCC were enrolled in this study. Their ages ranged from 26 to 64 years (median age, 49 years). Of these patients, three were diagnosed with stage I HCC, one with stage II and two with stage IV. All six patients were hepatitis B virus (HBV)-positive based on HBsAg test results. None of them were treated with chemotherapy or radiation prior to tumor resection. Adjacent normal tissue was at least 2 cm from the matched tumor tissue. All patients in this study provided written informed consent for sample collection and data analyses. This study was approved by the Ethics Committee of Beijing Ditan Hospital, Capital Medical University (Beijing, China) (Permit No. 2019044001).

### Antibodies and reagents

We used the following anti-CD146 antibodies: AA1, AA4 and AA98, generated in our library [10, 41], for FCM, immunoblotting or tumor therapy. Anti-CD146 (Cat. No. ab75769; Abcam, Cambridge, UK) was used for immunoblotting.

Other antibodies and reagents used were as follows: from BioLegend CNS, Inc. (San Diego, CA, USA), we obtained Brilliant Violet (BV) 570 anti-human CD3 (Cat. No. 300435), phycoerythrin (PE) anti-human CD146 (Cat. No. 302004), BV 785 anti-human CD3 (Cat. No. 344841), peridinin-chlorophyll-protein (PerCP)/cyanine 5.5 (Cy5.5) anti-human CD33 (Cat. No. 303414), allophycocyanin (APC)/Cy7 anti-human CD14 (Cat. No. 301820), PerCP anti-human CD163 (Cat. No. 333626), BV 421 anti-human CD15 (also known as stage-specific embryonic antigen-1 [SSEA-1]; Cat. No. 323039), BV 650 anti-human CD19 (Cat. No. 302237), PE/Cy7 anti-human HLA-DR (Cat. No. 307616), BV 711 anti-human CD27 (Cat. No. 302833), APC anti-human CD11b (Cat. No. 301310), BV 785 anti-human CD4 (Cat. No. 317441), BV 711 anti-human CD45 (Cat. No. 304050), PerCP/Cy5.5 anti-mouse CD3ε (Cat. No. 100327), BV 421 anti-mouse 1.1 (Cat. No. 108731), APC/Cy7 anti-mouse CD25 (Cat. No. 102025), Red Blood Cell (RBC) Lysis Buffer (10X; Cat. No. 420301), Alexa Fluor 700 anti-mouse CD45.2 (Cat. No. 109822), APC/Cy7 anti-mouse CD8a (Cat. No. 100714), fluorescein isothiocyanate (FITC) anti-mouse/human CD11b (Cat. No. 101206) and APC anti-mouse CD11c (Cat. No. 117310). Folin Ciocalteu Reagent (FcR) Mouse Blocking Reagent (Cat. No. 130-092-575) and FcR Blocking Reagent, Mouse (Cat. No. 130-092-575) were purchased from Miltenyi Biotech (Bergisch Gladbach, Germany). We obtained anti-mouse F4/80 antigen eFluor 450 (Cat. No. 48-4801-82), anti-mouse CD11b PerCP/Cy5.5 (Cat. No. 45-0112-82), IMJECT ALUM (Cat. No. 77161) and Accutase Enzyme Cell Detachment Medium (Cat. No. 00-4555-56) from eBioscience (Thermo Fisher Scientific, Waltham, MA, USA). Mouse (G3A1) mIgG_1_ Isotype Control (Cat. No. 5415S) and antibodies against phospho-p44/42 mitogen-activated protein kinase (*MAPK*; *Erk1/2*, threonine 202 [Thr202]/tyramine 204 [Tyr204]; Cat. No. 4370S), p44/42 *MAPK* (*Erk1/2*; Cat. No. 4695S), phospho-p38 *MAPK* ([Thr180/Tyr182]; Cat. No. 4511S), p38α *MAPK* (L53F8; Cat. No. 9228S), phospho–protein kinase B (Akt; serine 473 [Ser473]; Cat. No. 4060T), Akt (pan; 40D4; Cat. No. 2920S), phospho–NF-κB p65 (Cat. No. 3033S), NF-κB p65 (L8F6; Cat. No. 6956S), phospho-stress-activated protein kinase (*SAPK*)/*JNK* (Thr183/Tyr185; Cat. No. 4668S), *SAPK*/*JNK* (Cat. No. 9252S), phospho-*STAT3* (Tyr705; Cat. No. 9138S) and *STAT3* (Cat. No. 4904S) were purchased from Cell Signaling Technology (CST; Danvers, MA, USA). Anti–CD4-FITC (Cat. No. M10041-02B), CD8-PE (Cat. No. M10083-09B), CD69-FITC (Cat. No. M30691-02B) and CD4-APC (Cat. No. M10043-11A) were purchased from Tianjin Sungene Biotech Ltd. (Tianjin, China). We obtained Caspase-1 rabbit polyclonal antibody (pAb; Cat. No. A0964), TMEM176B rabbit pAb (Cat. No. A16118) and IL-1β rabbit pAb (Cat. No. A11369) from ABclonal Technology (Woburn, MA, USA). Anti-CD68 (KP1; Cat. No. ab955), anti-Arginase (Cat. No. ab124917), anti-IL-1β (Cat. No. ab254360), anti-NLRP3 (Cat. No. ab263899) and anti-Caspase-1 (Cat. No. ab179515) antibodies were purchased from Abcam. Beetle luciferin potassium salt (Cat. No. E1605) was purchased from Promega. We obtained BayK8644 (Cat. No. M5073) and the *STAT3* inhibitor (Cat. No. M3032) from AbMole (Polignac, France). The *JNK* inhibitor (Cat. No. S7508) was obtained from Selleck Chemicals (Houston, TX, USA). Bindarit (Cat. No. AF2838) and Maraviroc (Cat. No. UK-427857) were obtained from MedChemExpress (MCE, USA). CCL2, CCL3, CCL4, CXCL2 and CXCL5 ELISA kits (Cat. No. ED-20568-96T, ED-20427-96T, ED-21503-96T, ED-21268-96T, and ED-27806-96T, respectively) were purchased from LunChangShuoBiotech (Xiamen, China). We purchased in vivo mAb Anti-Mouse PD-1 (CD279; Cat. No. BE0146-25MG) and rat IgG_2a_ control (Cat. No. BE0089) from Bio X Cell (Lebanon, NH, USA).

### Mouse and human tumor tissue immune cell isolation

We isolated and excised tumor tissues from mice and ground them in phosphate-buffered saline (PBS) with calcium chloride and 0.5% bovine serum albumin (BSA). The suspensions were then centrifuged at 300 × *g* for 2 min to remove erythrocytes and free leukocytes. The pellets were resuspended in Dulbecco’s modified Eagle’s medium (DMEM) supplemented with 0.05% collagenase I and 30 U DNase and then shaken at 200 rpm and 37 °C for 45 min. After digestion, we allowed the cell suspension to settle for approximately 30 s. The supernatant was collected and washed three times with PBS at 2000 rpm for 5 min. We resuspended the pellet in RBC lysis buffer (eBioscience) and incubated it for 5 min to remove erythrocytes. The remaining cells were resuspended in FACS buffer (PBS with 0.01% BSA; BD Biosciences) at a concentration of 10^7^ cells/ml. After incubating them with Fc Blocker (BD Biosciences), we stained the cells with conjugated antibodies for 45 min at 4 °C and then washed them twice.

We isolated human tumor tissue immune cells per the instructions of a Tumor Dissociation Kit (Miltenyi). Briefly, tissues were weighed, cut into small pieces of 2–4 mm and then transferred into a gentleMACS C Tube (Miltenyi) containing the enzyme mix. We dissociated the tissue mix using a gentleMACS Dissociator (Miltenyi), resuspended the sample and applied the cell suspension to a MACS SmartStrainer (70 μm; Miltenyi) placed in a 50-ml tube. Cells were washed with 20 ml Roswell Park Memorial Institute (RPMI) 1640 medium and then centrifuged at *300 × g* for 7 min. We resuspended cells as required for further applications.

### BMDM cell culture

Primary bone marrow-derived macrophages (BMDMs) were prepared as previously described [14]. Briefly, we flushed tibias and femurs isolated from 6- to 8-week-old CD146 WT and CD146-KO mice to obtain bone marrow cells. After RBC lysis, the remaining cells were harvested and grown in complete DMEM culture medium supplemented with 15% L929-conditioned media for 7 days. We cultured differentiated BMDMs in complete DMEM and used them for the following experiments.

### Tumor cell-conditioned medium (TCM) preparation and BMDM stimulation

For TCM preparation, Hepa1-6 or MC38 tumor cells were cultured in their respective medium for 2 days. The cultured medium was collected and centrifuged at 4000 rpm for 10 min and stored at −20 °C. For BMDM stimulation, TCM was diluted 1:1 with complete DMEM culture medium and then added to BMDMs. The stimulation medium was changed daily.

### Protein extraction and in-solution digestion

BMDMs were cultured in complete culture medium or stimulated with Hepa1-6–conditioned medium for 72 h. We washed cells twice with PBS and harvested them by scraping in ice-cold radioimmunoprecipitation assay (RIPA) buffer supplemented with protease inhibitor cocktail (Roche). Cell lysates were centrifuged at 16,000 × *g* for 15 min at 4 °C to remove cell debris, after which we collected the supernatants and stored them at −80 °C until use. Cell lysates were denatured via filter-aided sample preparation (FASP) with minor modifications [42]. Briefly, we transferred the lysates to Amicon 0.5-mL ultrafiltration units (EMD Millipore, Burlington, MA USA) and exchanged the buffer three times with uric acid (UA) buffer (8 M urea, 0.1 M Tris; pH 8.5). Protein samples were reduced with 10 mM dithiothreitol at 37 °C for 1 h and then alkylated with 50 mM iodoacetamide at room temperature in the dark for 1 h. We then exchanged the buffer of the denatured protein samples three times with UA buffer and twice with 50 mM tetraethylammonium bromide (TEAB). Proteins were recovered by reverse centrifugation at 1000 × *g* for 2 min and resolved in 50 mM TEAB. We determined protein concentrations in triplicate using the bicinchoninic acid (BCA) method (Pierce [Thermo Scientific]) per the manufacturer’s instructions. One hundred micrograms of each sample was digested with trypsin at 37 °C overnight. We desalted all peptide samples using self-packed reverse-phase columns.

### Eight-plex isobaric tag for relative and absolute quantitation labeling

Per the manufacturer’s instructions, we labeled peptides with the 8-plex iTRAQ reagent. Briefly, peptides derived from the CD146^+/+^ macrophage cells treated with or without Hepa were labeled with iTRAQ tags 113/114 (two independent biological experiments), and 117/118, while peptides derived from CD146^−/−^ macrophage cells treated with or without Hepa were labeled with iTRAQ tags 115/116, and 119/121, respectively. After labeling, samples were combined and acidified, and the peptides were desalted using self-packed reverse-phase columns.

### High-pH prefractionation

After desalting them using C18 SPE cartridges (Waters Corp., Milford, MA, USA), we fractionated the labeled peptides using a YMC-Triart C18 basic reverse-phase liquid chromatography column (250 × 4.6 mm; 5-μm particles; YMC America, Devens, MA, USA). Eluates were collected every 75 s, and a total of 65 fractions were obtained. We discarded the first five fractions because most of the excess labeling reagent was eluted during these fractions. The remaining 60 fractions were reduced to 15 by merging fractions 1, 16, 31 and 46; fractions 2, 17, 32 and 47; and so forth. Then, we dried all fractions in a vacuum concentrator (LABCONCO, Kansas, MO, USA) and stored them at −20 °C until further analysis.

### Liquid chromatography–tandem mass spectrometry analysis

We collected all mass spectrometry (MS) data on a Q-Exactive mass spectrometer coupled with an Easy-nLC system (both Thermo Fisher). Each fraction of tryptic peptides was resuspended in 0.1% formic acid (FA) and separated on a C18 column (75 µm × 20 cm) packed with Reprosil-Pur C18 AQ particles (3.0 μm; Dr. Maisch HPLC GmbH; Ammerbuch, Germany) with solvents A (0.1% FA) and B (acetonitrile [ACN]/0.1% FA) at a flow rate of 300 nl/min with a 78-min gradient: from 5% to 8% B in 8 min, from 8% to 22% B in 50 min, from 22% to 32% B in 12 min, from 32% to 95% B in 1 min, and then maintenance of B at 95% for 7 min.

We then used Q-Exactive for MS analysis in data-dependent acquisition mode. Each MS1 spectrum was obtained at 70,000 high resolution (*m*/*z* 200) at 300–1600 *m*/*z*. The automatic gain control (AGC) target value was 3E6 for a maximum filling time of 60 ms. We selected the top 20 most abundant precursor ions with a 2.0-*m*/*z* isolation window and fragmented them with a normalized collision energy of 27%. Tandem MS (MS–MS) spectra were acquired at 17,500 resolution (*m*/*z* 200) with a target value of 50,000 ions over a maximum injection time of 60 ms by setting up an isolation window of 2.0 *m*/*z* and dynamic exclusion time of 50 s.

### Protein identification and quantification

Raw data were processed using Proteome Discoverer software v2.2 (Thermo Fisher), the SEQUEST HT search engine (Eng, McCormack & Yates, 1994) and a human database (including 20,385 proteins downloaded in August 2018) appended with known contaminants. We selected trypsin as the enzyme and allowed only two missed cleavages. The precursor mass tolerance was 10 ppm, and the product ion tolerance was 0.02 Da. Cysteine carbamidomethylation and iTRAQ 8-plex labeling at the N-terminus and lysine residues were set as fixed modifications, while methionine oxidation and N-terminal acetylation were set as variable modifications. We used the percolator algorithm for false discovery rate (FDR) analysis. Peptides with FDR < 1% were set as high-confidence peptides. For protein quantification, we set filters via normalization with protein median and used only unique peptides.

### Assay of BMDM phagocytosis of tumor cells

The tumor cell lines Hepa1-6 and EL4 were obtained from ATCC. Hepa1-6 or EL4 cells were labeled with the cytosolic dye 5′-carboxyfluorescein succidyl ester (CFSE). We seeded 5 × 10^5^ BMDMs in 6-well plates and serum starved them for 4–6 h, after which we added 1 × 10^6^ CFSE-labeled tumor cells and cocultured them for 2 h. After free cells were washed, macrophages that had phagocytized CFSE-labeled cells were counted.

### Tumor models

The MC38 and Raw264.7 cell lines were obtained from ATCC. To establish our xenograft model, we subcutaneously injected 1 × 10^6^ Hepa1-6 cells or 2 × 10^5^ MC38 or EL4 cells into the flanks of M-WT or M-KO mice. Tumor sizes were measured every 3 days, and tumor volume was calculated according to the Formula *V* = ½ × length × width × width. For tumor treatment, we intraperitoneally (i.p.) injected mice with AA98 (10 mg/kg) alone or combined with BayK8644 (1 mg/kg, 1×/day), *STAT3* inhibitor (10 mg/kg) or anti-PD-1 (10 mg/kg) 2×/week from Day 7 after tumor inoculation.

To establish our DEN-induced primary liver cancer model, we injected 14-day-old male mice i.p. with DEN (1 mg/kg) and then with CCl_4_ (0.2 ml/kg) i.p. 2×/week starting at 8 weeks of age. Mice were sacrificed at 22 weeks of age to analyze tumor development.

To establish our macrophage and tumor cell coinjection model, we subcutaneously injected 6 × 10^6^ BMDMs or 2 × 10^5^ Raw264.7 cells mixed with 2 × 10^5^ EL4 cells into the flanks of female C57BL/6 J or EGFP-transgenic mice or Rag1^−/−^ mice. Tumor sizes were measured every 3 days.

### MDSC transmigration assay

The mouse endothelial cell line SEND-1 was obtained from ATCC. We isolated spleen MDSCs from EL4 tumor-bearing mice. SEND-1 cells (5 × 10^5^) were seeded into the apical chamber of a 96-well Transwell plate (Corning Labware Products Inc., Corning, NY, US) for 100% confluence. M-WT or M-KO macrophages were cultured in the lower chamber. We isolated fresh spleen MDSCs (1 × 10^6^) from EL4 tumor-bearing mice and added them to the apical chamber of the Transwell plate. After 12 h, we counted the MDSCs in the lower chamber.

### Luminex multiplex analysis of serum proinflammatory factors

We collected 50 μl sera from DEN-induced 22-week-old M-WT and M-KO mice and characterized profiles of proinflammatory factors using the Cytokine & Chemokine 36-Plex Mouse ProcartaPlex Panel 1A per the manufacturer’s instructions.

### Quantitative reverse transcription polymerase chain reaction

Total RNA was isolated using TRIzol reagent (Invitrogen Corp., Carlsbad, CA, USA), reverse transcribed into cDNA using random primers and then subjected to qRT-PCR using ChamQ Universal SYBR qPCR Master Reagent (Vazyme Biotech Co., Ltd, Nanjing, China) per the manufacturer’s instructions. The primers used are listed in Table S3.

### Statistical analysis

We used GraphPad Prism software 8.3.1 (GraphPad Software, Inc., San Diego, CA, USA) for statistical analysis. All bar graphs show the mean ± standard error of the mean (SEM), as indicated in each legend. Differences between groups were examined by Student’s *t* test or one-way analysis of variance (ANOVA), followed by Bonferroni’s correction or two-way ANOVA with a multiple-comparison test for comparison of means, as indicated in each legend. *P* values < 0.05 were considered statistically significant.

## Supplementary information

Uncut gel for Figures

Supplemental Fig1-4

Table S1

Table S2

Table S3
