# Supplementary material for: A subpopulation of CD146+ macrophages enhances antitumor immunity by activating the NLRP3 inflammasome
Source: Cell Mol Immunol. 2023 Jun 12;20(8):908–23. doi: 10.1038/s41423-023-01047-4 (PMC10387481; doi:10.1038/s41423-023-01047-4)

Uncut gel for Figure 2

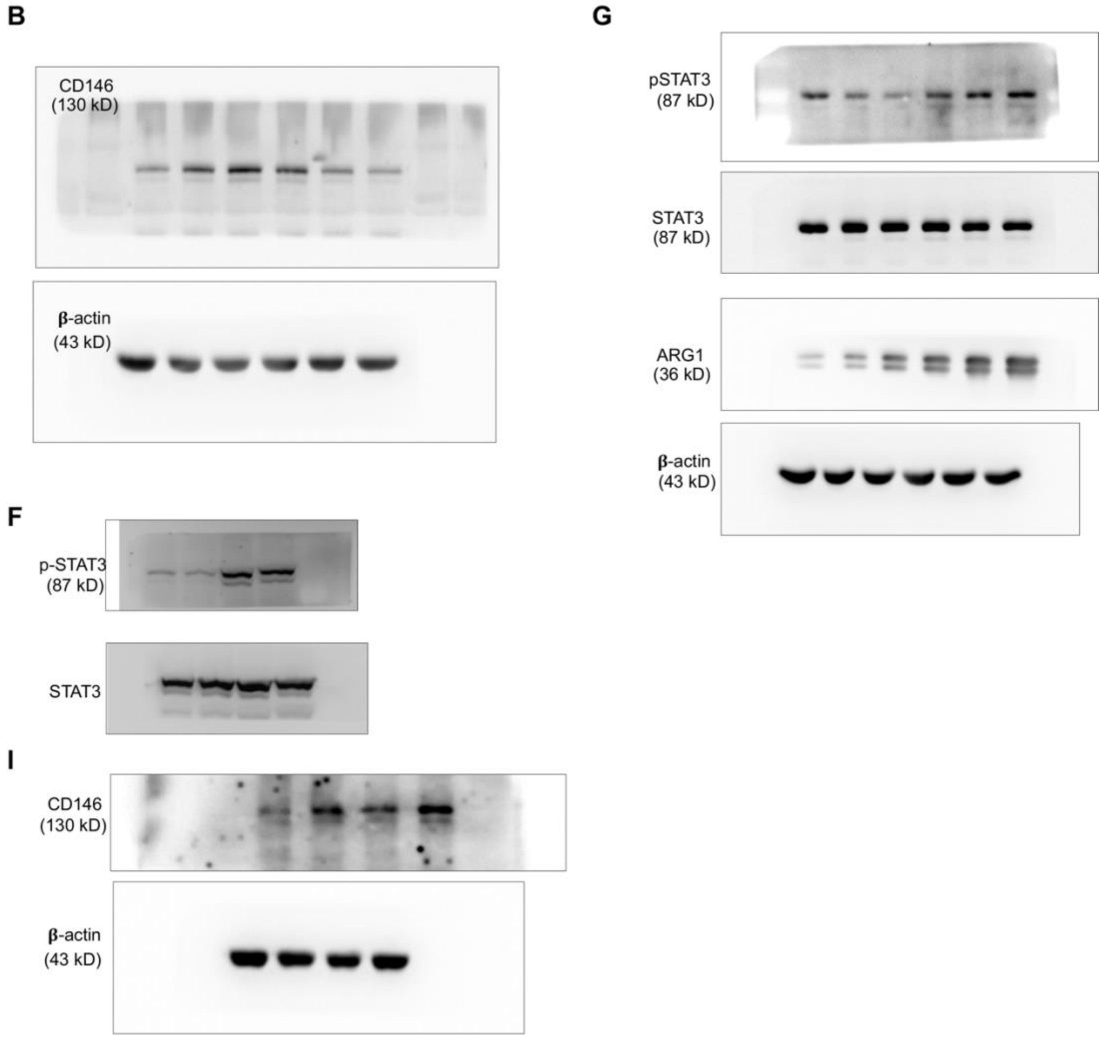

Uncut gel for Figure 6

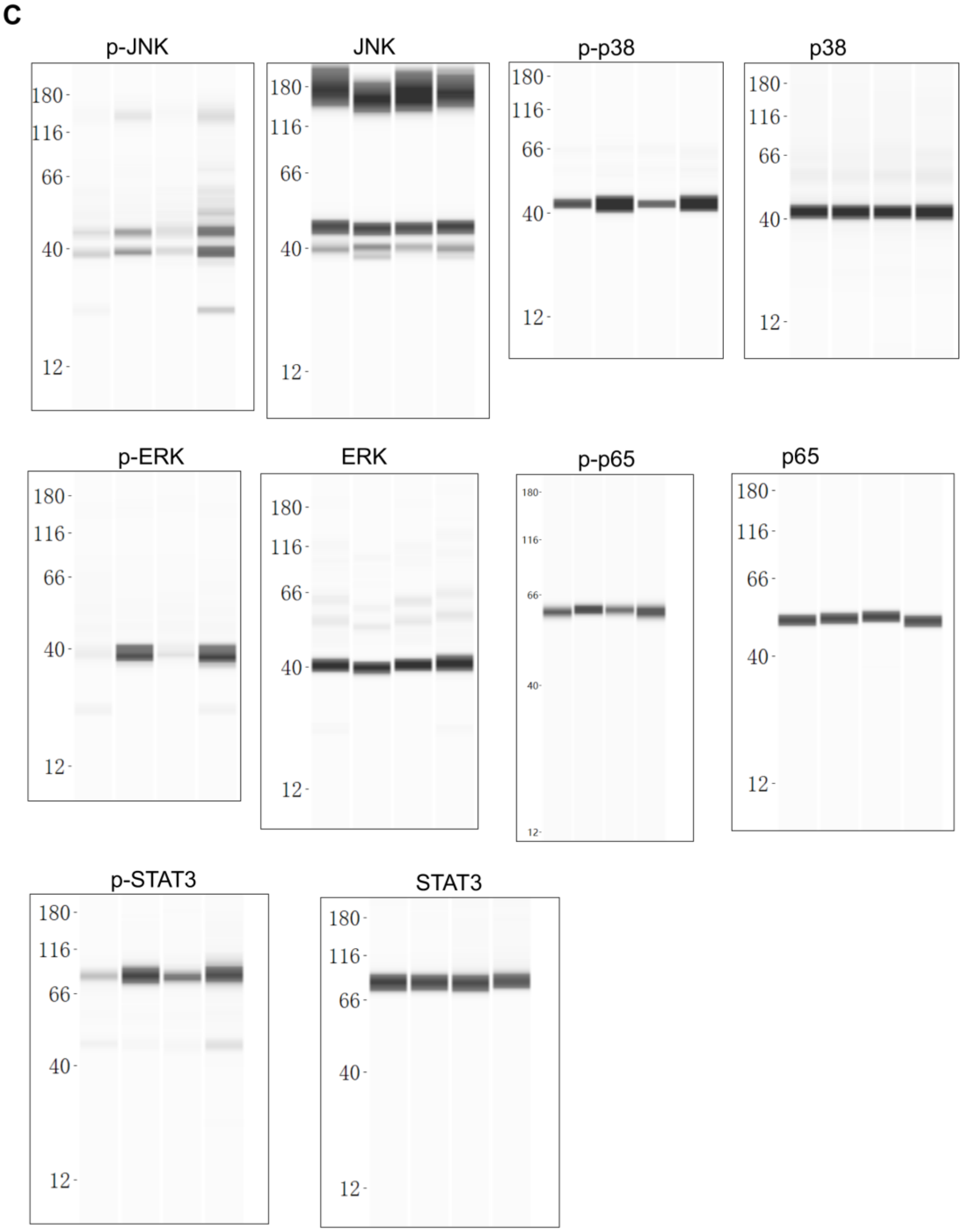

Uncut gel for Figure 7

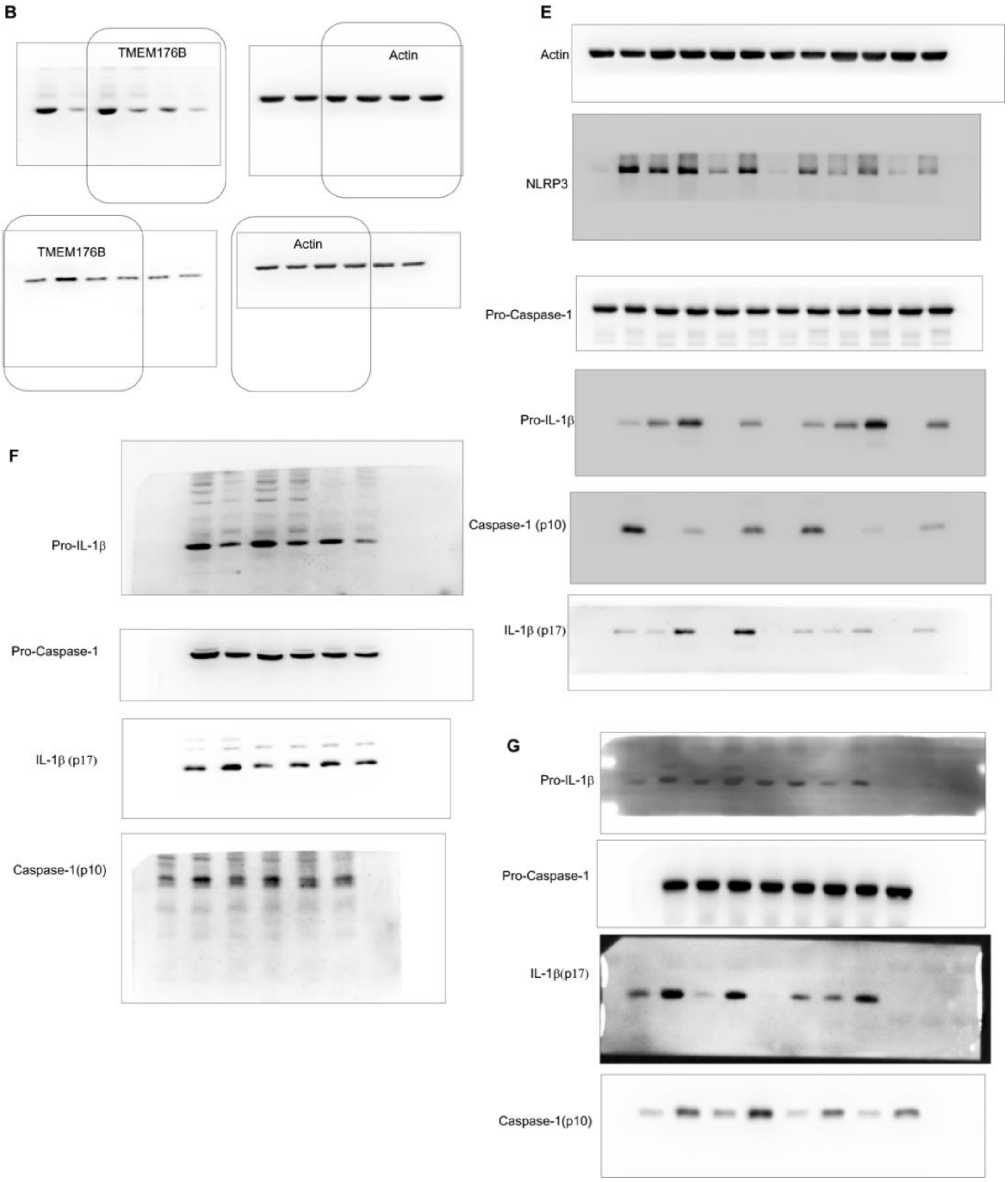

Uncut gel for Figure 8

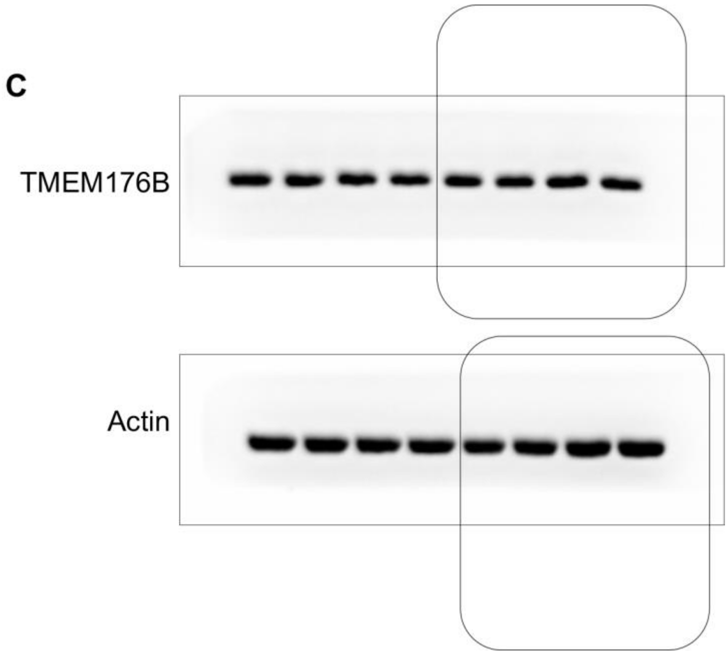

Uncut gel for Figure S2

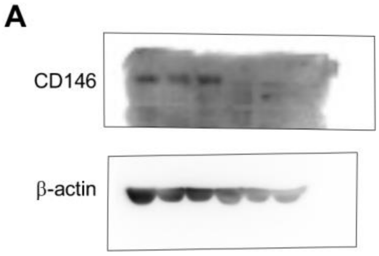

Supplement: Supplementary file 1 — Uncut gel for Figures [file 41423_2023_1047_MOESM1_ESM.pdf]
