# Supplementary material for: A subpopulation of CD146+ macrophages enhances antitumor immunity by activating the NLRP3 inflammasome
Source: Cell Mol Immunol. 2023 Jun 12;20(8):908–23. doi: 10.1038/s41423-023-01047-4 (PMC10387481; doi:10.1038/s41423-023-01047-4)
Supplement: Supplementary file 2 — Supplemental Fig1-4 [file 41423_2023_1047_MOESM2_ESM.docx]

**CD146 identifies a subpopulation of macrophages that enhances antitumor immunity by activating the inflammasome**

Lin Jing^1,#^，Yunhe An^2,#^, Tanxi Cai^3,4,#^, Jianquan Xiang^1,5^, Baoming Li^2^, Jiang Guo^6^, Xinran Ma^1,5^, Ling Wei^2^, Yanjie Tian^2^, Xiaoyan Cheng^2^, Xuehui Chen^1^, Zheng Liu^1^, Jing Feng^1^, Fuquan Yang^1,4,5 ,*^, , Xiyun Yan^1,5,7,*^ Hongxia Duan^1,*^

^1^Key Laboratory of Protein and Peptide Pharmaceutical, Institute of Biophysics, Chinese Academy of Sciences, Beijing 100101, China

^2^ Institute of Analysis and Testing, Beijing Academy of Science and Technology (Beijing Center for Physical and Chemical Analysis), No. 7 Fengxian Middle Street, Haidian District, Beijing 100094, China

^3^ Sino-Danish College，University of Chinese Academy of Sciences, Beijing 100049, China

^4^ Laboratory of Proteomics, Institute of Biophysics, Chinese Academy of Sciences, Beijing, China

^5^ College of Life Sciences, University of Chinese Academy of Sciences, Beijing 100049, China

^6^ Department of Interventional Oncology, Beijing Ditan Hospital, Capital Medical University, No. 8 Jingshun East Street, Chaoyang District, Beijing 100015, China

^7^Joint Laboratory of Nanozymes in Zhengzhou University, School of Basic Medical Sciences, Zhengzhou University, Zhengzhou 450001, China.

^#^These authors contributed equally to this work.

*Correspondence:

Fuquan Yang, E-mail: fqyang@ibp.ac.cn. Tel.: +86 10 64888581.

Xiyun Yan, E-mail: [yanxy@ibp.ac.cn](mailto:yanxy@ibp.ac.cn). Tel.: +86 10 64888583. Fax: +86 10 64888584.

Hongxia Duan, E-mail: cherryshoen@ibp.ac.cn. Tel.: +86 10 64888580. Fax: +86 10 64888584.

**Running title: CD146^+^ macrophages enhance antitumor immunity**

**Supplementary Figures**

**
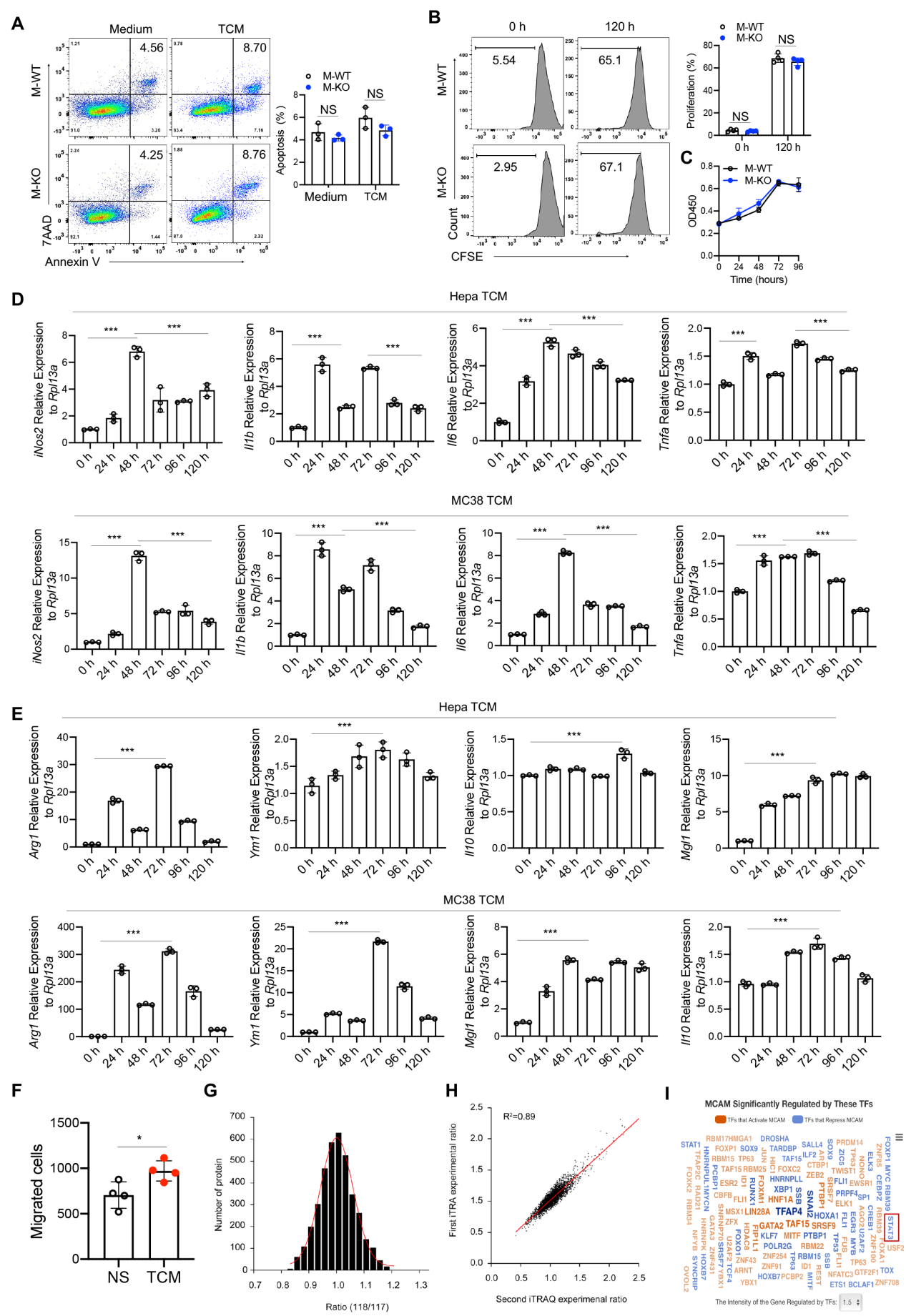
**

**Figure S1. The expression of CD146 on macrophages is controlled by the TME.** **(A)** FACS analysis of BMDM apoptosis under TCM stimulation for 48 h. **(B, C)** FACS (B) or CCK8 (C) analysis of BMDM proliferation under TCM stimulation for the indicated time. **(D, E)** *iNos, Il1b, Il6 and Tnfa* (D) or *Arg1, Ym1, Mgl1, and Il10* (E) gene expression in BMDMs stimulated with TCM for the indicated time. **(F)** BMDM migration assay. NS, basal culture medium; TCM, tumor cell–conditioned medium. **(G)** Histogram of distribution of the ratios of two biological replicates to determine cutoff values for protein level change. **(H)** Scatter plots of protein data from two biologically independent iTRAQ experiments. Ratios of iTRAQ signals from one experiment were plotted against those from a second experiment. The fit to the line *y* = *x* with *r*-square values (*r*^2^) was 0.89, indicating that our proteomics approach was highly reproducible. **(I)** TFs for regulating CD146 expression as shown by TF knockdown analysis. Each symbol represents an individual experiment (A, B–F). A one-way ANOVA (A, B, D, E) or a two-tailed *t* test (F) was performed. A one-way ANOVA (C) was performed. Data are shown as the mean ± SEM. NS, no significance, **P* < 0.05, ***P* < 0.01, ****P* < 0.001.


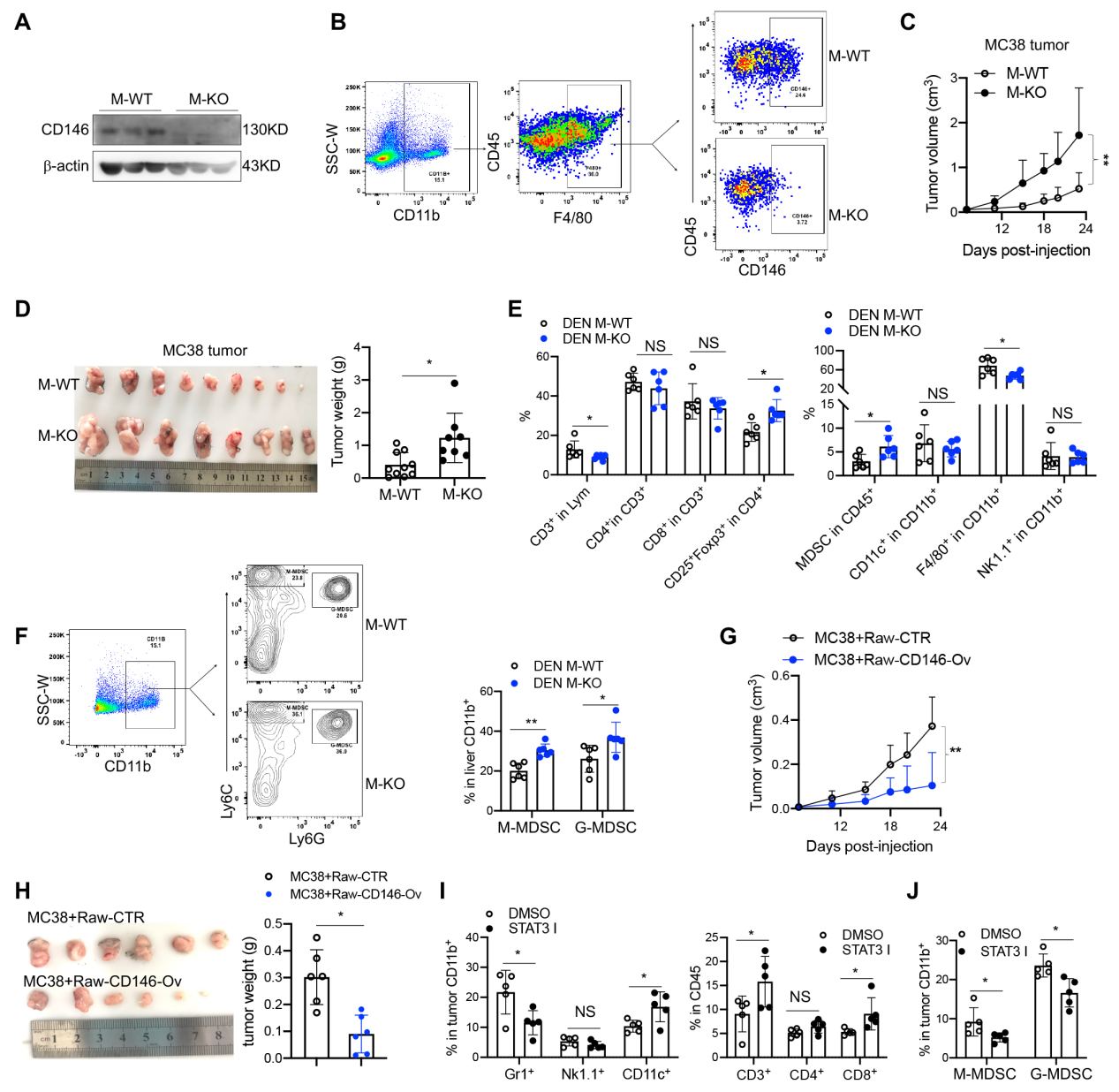


**Figure S2. CD146 on macrophages inhibited tumor development.** **(A, B)** WB (A) and FACS (B) analysis of CD146 expression on M-WT or M-KO macrophages. **(C, D)** MC38 tumor development (C) and tumor weight (D) in M-WT and M-KO mice (n = 10). **(E, F)** FACS analysis indicated different subpopulations in DEN-induced HCC tumors from M-WT or M-KO mice (n = 6). **(G, H)** Co-injection MC38 tumor model development (G) and tumor weight (H) (n = 6). **(I, J)** FACS analysis of Gr1^+^, NK1.1^+^ and CD11c^+^cell percentages in the CD11b^+^ population (left panel, I), or CD3^+^, CD4^+^, and CD8^+^ in the CD45^+^ population (right panel, I) or the M-MDSC and G-MDSC population (J) in *STAT3*inhibitor–treated mice and control mice (n = 5). Each symbol represents an individual mouse (D, E, F, H, I, J). A two-way ANOVA with a multiple-comparison test (C, G) or a two-tailed *t* test (D, H) or one-way ANOVA (E, F, I, J) was performed. Data are shown as the mean ± SEM. NS, no significance, **P* < 0.05, ***P* < 0.01, ****P* < 0.001.


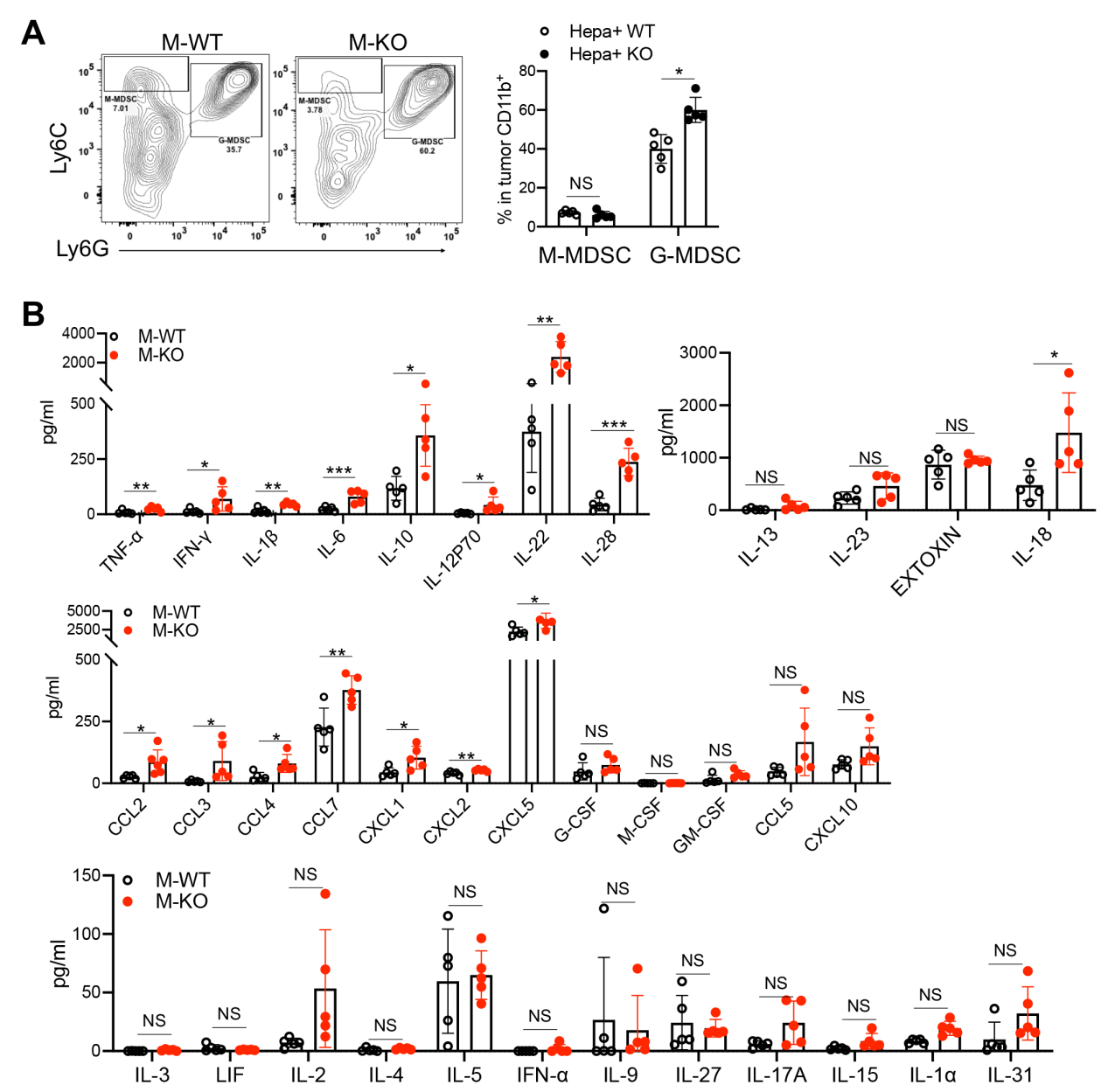


**Figure S3. CD146 deletion on macrophages promoted the recruitment of MDSCs. (A)** FACS analysis of M-MDSC and G-MDSC in the co-injection tumor model (n = 5)**. (B)** Luminex multiplex assays of sera from the DEN-induced liver cancer model (n = 5). A one-way ANOVA was performed. Data are shown as the mean ± SD. NS, no significance,**P* < 0.05, ***P* < 0.01, ****P* < 0.001.

**
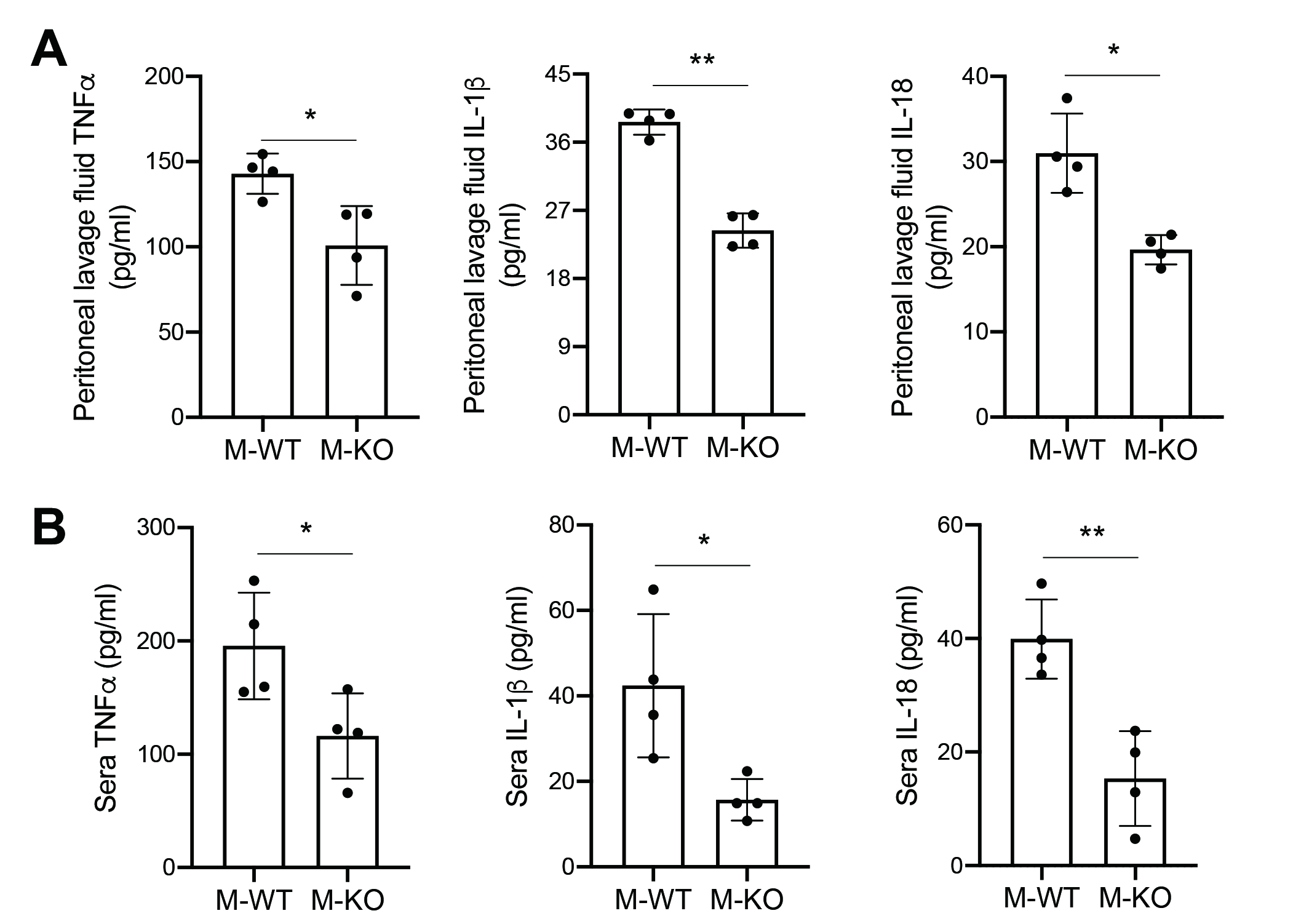
**

**Figure S4.** **CD146 on macrophages promoted lipopolysaccharide (LPS)-induced inflammasome activation.** (A-B) Double antibody sandwich enzyme-linked immunosorbent assay for peritoneal lavage fluid (A) or sera (B) TNF-α, IL-1β and IL-18 levels. A two-tailed *t* test (A, B) was performed. Data are shown as the mean ± SEM. **P* < 0.05, ***P* < 0.01, ****P* < 0.001.
