# Supplementary material for: A subpopulation of CD146+ macrophages enhances antitumor immunity by activating the NLRP3 inflammasome
Source: Cell Mol Immunol. 2023 Jun 12;20(8):908–23. doi: 10.1038/s41423-023-01047-4 (PMC10387481; doi:10.1038/s41423-023-01047-4)
Supplement: Supplementary file 5 — Table S3 [file 41423_2023_1047_MOESM5_ESM.docx]

**Table S3. Primers used in this study**

| **Gene name** | **Forward (5′ to 3′)** | **Reverse (5′ to 3′)** |
| --- | --- | --- |
| ***mCD146*** | AGTCCTCACACCAGAGCCAA | CTCTTACGAGTCGGGGGCA |
| ***mArg1*** | CCACAGTCTGGCAGTTGGAAG | GGTTGTCAGGGGAGTGTTGATG |
| ***miNos*** | GCGCTCTAGTGAAGCAAAGC | GGCCTTGTGGTGAAGAGTGT |
| ***mIl1b*** | AACAGGCTGCTCTGGGATTC | AGATTCGTAGCTGGATGCCG |
| ***mMgl1*** | CAGAATCGCTTAGCCAATGTGG | *TCCCAGTCCGTGTCCGAAC* |
| ***mIl10*** | GCTCCAAGACCAAGGTGTCT | *CGGAGAGAGGTACAAACGAGG* |
| ***mTnfa*** | CCCAGGGACCTCTCTCTAATCA | *AGCTGCCCCTCAGCTTGAG* |
| ***mIl6*** | GCCTTCTTGGGACTGATGCT | *CTGCAAGTGCATCATCGTTGT* |
| ***mRpl13a*** | GAGGTCGGGTGGAAGTACCA | TGCATCTTGGCCTTTTCCT |
| ***mTmem176b*** | ACTCCAGCTAGAATTGCCACAG | CATCAGCATCCACATCCACC |
| ***mCcl2*** | TTAAAAACCTGGATCGGAACCAA | GCATTAGCTTCAGATTTACGGGT |
| ***mCcl3*** | TTCTCTGTACCATGACACTCTGC | CGTGGAATCTTCCGGCTGTAG |
| ***mCcl4*** | TTCCTGCTGTTTCTCTTACACCT | CTGTCTGCCTCTTTTGGTCAG |
| ***mCcl5*** | GCTGCTTTGCCTACCTCTCC | TCGAGTGACAAACACGACTGC |
| ***mCcl7*** | GCTGCTTTCAGCATCCAAGTG | CCAGGGACACCGACTACTG |
| ***mCxcl1*** | CTGGGATTCACCTCAAGAACATC | CAGGGTCAAGGCAAGCCTC |
| ***mCxcl2*** | CCAACCACCAGGCTACAGG | GCGTCACACTCAAGCTCTG |
| ***mCxcl3*** | TGCACCCAGACAGAAGTCAT | GACACCGTTGGGATGGATCG |
| ***mCxcl5*** | GTTCCATCTCGCCATTCATGC | GCGGCTATGACTGAGGAAGG |
| ***mCxcl9*** | GGAGTTCGAGGAACCCTAGTG | GGGATTTGTAGTGGATCGTGC |
